# How the Support Defines Properties of 2D Metal–Organic Frameworks: Fe-TCNQ on Graphene versus Au(111)

**DOI:** 10.1021/jacs.3c13212

**Published:** 2024-01-22

**Authors:** Zdeněk Jakub, Azin Shahsavar, Jakub Planer, Dominik Hrůza, Ondrej Herich, Pavel Procházka, Jan Čechal

**Affiliations:** † CEITEC−Central European Institute of Technology, Brno University of Technology, Purkyňova 123, Brno 61200, Czech Republic; ‡ Institute of Physical Engineering, Faculty of Mechanical Engineering, Brno University of Technology, Technická 2896/2, Brno 61200,Czech Republic

## Abstract

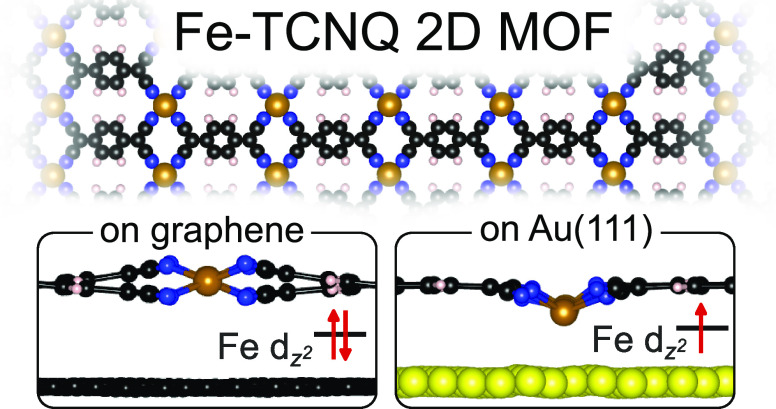

The functionality of 2D metal–organic frameworks (MOFs) is crucially dependent on the local environment of the embedded metal atoms. These atomic-scale details are best ascertained on MOFs supported on well-defined surfaces, but the interaction with the support often changes the MOF properties. We elucidate the extent of this effect by comparing the Fe-TCNQ 2D MOF on two weakly interacting supports: graphene and Au(111). We show that the Fe-TCNQ on graphene is nonplanar with iron in quasi-tetrahedral sites, but on Au(111) it is planarized by stronger van der Waals interaction. The differences in physical and electronic structures result in distinct properties of the supported 2D MOFs. The d_*z*^2^_ center position is shifted by 1.4 eV between Fe sites on the two supports, and dramatic differences in chemical reactivity are experimentally identified using a TCNQ probe molecule. These results outline the limitations of common on-surface approaches using metal supports and show that the intrinsic MOF properties can be partially retained on graphene.

## Introduction

Two-dimensional metal–organic frameworks (2D MOFs) show great potential for applications in single-atom catalysis, spintronics, or high-density data storage.^1−8^ The properties of 2D MOFs crucially depend on the local environment and electronic structure of the metal atoms, and these fine details are best ascertained on systems synthesized on metal surfaces in ultrahigh vacuum.^9,10^ However, the interaction with the metal support makes these systems difficult to compare to the free-standing models commonly screened by density functional theory (DFT) computations,^11−14^ or to systems prepared by traditional synthesis methods relevant for applications.^15−17^ Ways to minimize the support–MOF interaction are actively sought to bridge this gap, but it is not always easy to determine what is “weak enough” for a given application. Here, we show how the support–MOF interaction affects the structural and electronic properties of supported 2D MOFs and how this ultimately affects the crucial parameters for applications in single-atom catalysis and spintronics. We demonstrate that 2D MOFs synthesized on an inert graphene support present suitable models for studies of the intrinsic MOF properties, which are difficult to achieve on metal supports.

By a combined experimental and theoretical approach, we studied atomically defined Fe-TCNQ 2D metal–organic frameworks supported on two weakly interacting supports: graphene/Ir(111) and Au(111). Au(111) belongs to the most popular supports for on-surface synthesis^10,18−21^ and is considered weakly interacting as the molecules adsorbed atop Au(111) generally show low binding energies, and their properties are less perturbed than on other common metal substrates.^22,23^ Nevertheless, chemical bonds to the Au substrate can be formed, and Au atoms can be easily shifted around upon molecular adsorption.^19,21,24−26^ In contrast, epitaxial graphene/Ir(111) is chemically inert and structurally stable, and even the van der Waals (vdW) interaction between the graphene and adsorbate is weaker because of the low electron density and low polarizability of the graphene support. In what follows, we demonstrate that the weaker vdW interaction with the graphene support plays a decisive role in the structural stabilization of an ordered nonplanar configuration of a supported Fe-TCNQ 2D metal–organic network, in which the Fe atoms adopt quasi-tetrahedral coordination. A similar structure is only metastable on Au(111), where the vdW interaction draws Fe atoms toward the Au support. The structural distortions of the Fe-TCNQ layer and the different work functions of the two supports then significantly change the position of the Fe d-orbitals with respect to the Fermi level, which dramatically changes the predicted reactivity of these systems.

## 2 Results and Discussion

**Figure 1:**
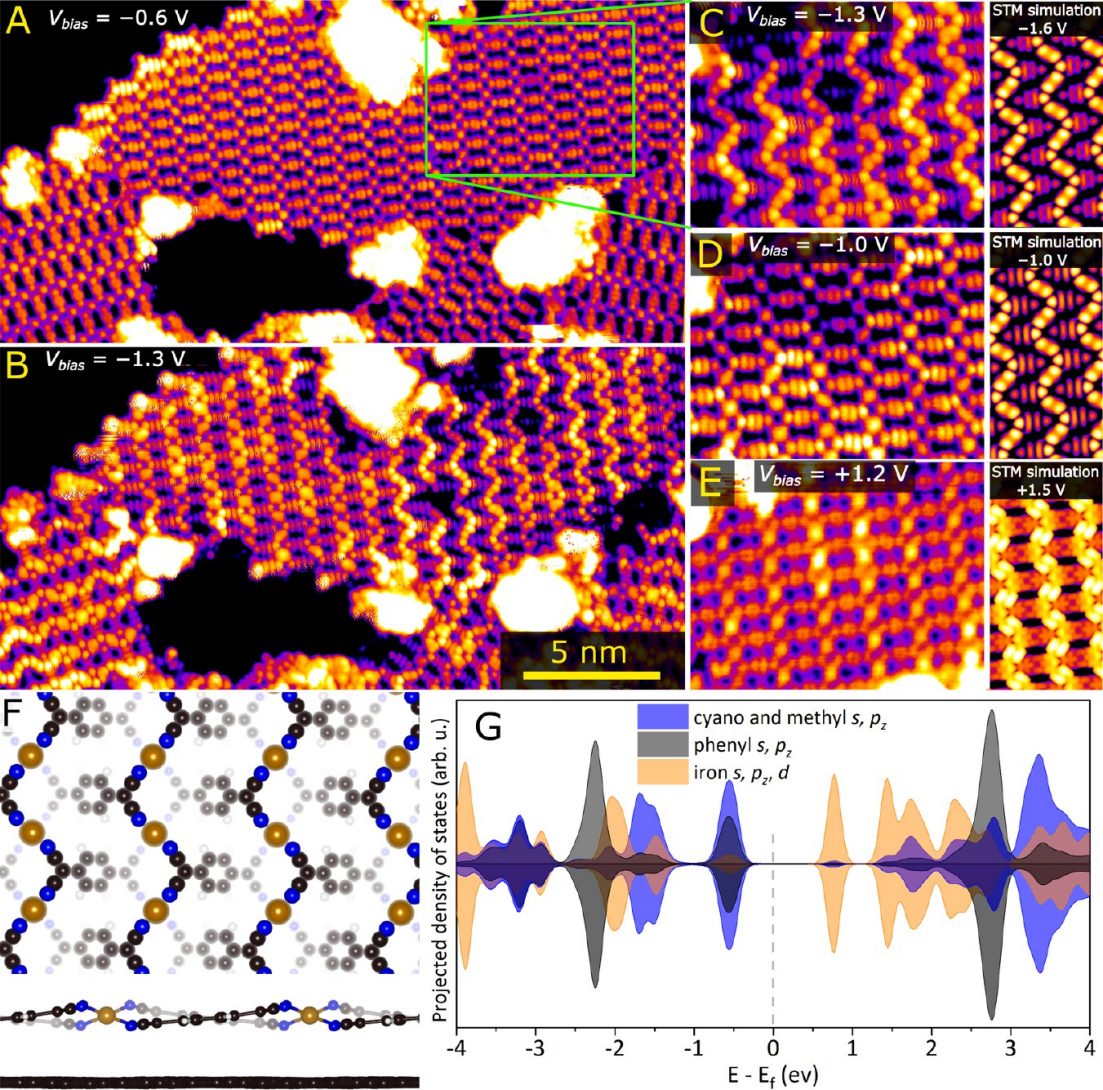
Room-temperature scanning tunneling microscopy images of the graphene-supported Fe-TCNQ 2D MOF taken at different sample bias settings compared to computational models. (A) The Fe-TCNQ looks planar and homogeneous at sample bias *V* = −0.6 V. (B) A distinct zigzag pattern is observed at sample bias *V* = −1.3 V. (C–E) Detailed STM images taken at the same spot with different sample bias settings compared to STM simulations. (F) Top and side views of the relaxed graphene-supported Fe-TCNQ 2D MOF. Depth-shading was used to highlight the height corrugation of the system. The simulated unit cell is shown in Figure S4 in the SI and contains four Fe_1_(TCNQ)_1_ units. (G) Projected density of states plot showing the DOS located at the cyano- and methyl groups (blue), phenyl ring (gray), and iron (brown).

Figure 1 shows room-temperature scanning tunneling microscopy (STM) images of Fe-TCNQ synthesized on graphene following the protocol described in the Methods section and ref (27). Prior to STM measurements, the sample was annealed to 480 °C for 30 min, which led to a high structural quality of the Fe-TCNQ networks but also partial decomposition near domain boundaries, resulting in the presence of a few Fe clusters. The Fe-TCNQ 2D MOF looks homogeneous in an image taken at sample bias *V* = −0.6 V, shown in Figure 1A. This appearance is consistent with commonly reported models for metal-TCNQ MOFs, where the molecules lie in plane and the metal center resides in a square-planar environment. However, in images taken at a *V* = −1.3 V on the same spot (Figure 1B), the Fe-TCNQ network features a distinctive zigzag pattern on up to 50% of the MOF area. In this pattern, a part of the TCNQ molecules appears significantly brighter than the rest (large-scale images taken at different spots are shown in Figures S1–S3 in the SI). The zigzag appearance is strongly dependent on the bias voltage, as shown in detailed images in Figure 1C–E.

We interpret this observation with the help of DFT + U calculations. These show that a nonplanar structure of Fe-TCNQ is the most stable both in the gas phase and on graphene. In this model, shown in Figure 1F, the Fe atoms do not reside in a square-planar environment but adopt a distorted tetrahedral geometry. This is accommodated by tilting the TCNQ molecules by ≈8° from the graphene support plane, as shown in Figure 1F. In the gas phase models (without the graphene support), the Fe sites are almost perfectly tetrahedral, and the TCNQ tilt is 14°. Within our computational setup, the expected planar structure does not present even a local energetic minimum, but we can calculate an energy reference by relaxing a planar model while keeping the *z* positions of the atoms constant. Doing this, we found that the nonplanar model is favored by 350 meV per Fe_1_(TCNQ)_1_ unit in the gas phase and remains preferred by 140 meV per Fe_1_(TCNQ)_1_ on graphene. The energy differences between the different models in the gas phase and on graphene are summarized in Tables ST2 and ST3 in the SI. We note that the preference for the nonplanar configuration in the gas phase is independent of the Hubbard U parameter used for Fe (between 3 and 6 eV), the initial spin multiplicity, and the choice of DFT functional between optPBE-vdW and PBE-D3.

The pronounced zigzag pattern is well reproduced by constant-current STM simulations based on the tilted-TCNQ model and Tersoff–Hamann approach (side panel of Figure 1C), confirming that the pattern is formed by the methyl and cyano groups that reside the highest above the support. The strong dependence of the zigzag pattern visibility on the experimental scanning parameters is qualitatively explained by the projected density of states plot shown in Figure 1G: whereas the states at −1.5 eV are dominated by orbitals located at the methyl and cyano groups, the states at −0.7 eV feature a much higher share of orbitals located at the phenyl ring. Therefore, as the negative sample bias voltage gets closer to 0 V, the phenyl rings get more pronounced, resulting in a less discernible zigzag pattern. This is qualitatively confirmed by the STM simulation shown in Figure 1D. At a positive sample bias of +1.2 V (Figure 1E), the experimental STM image shows pronounced Fe sites, in agreement with the PDOS plot and STM simulation.

**Figure 2:**
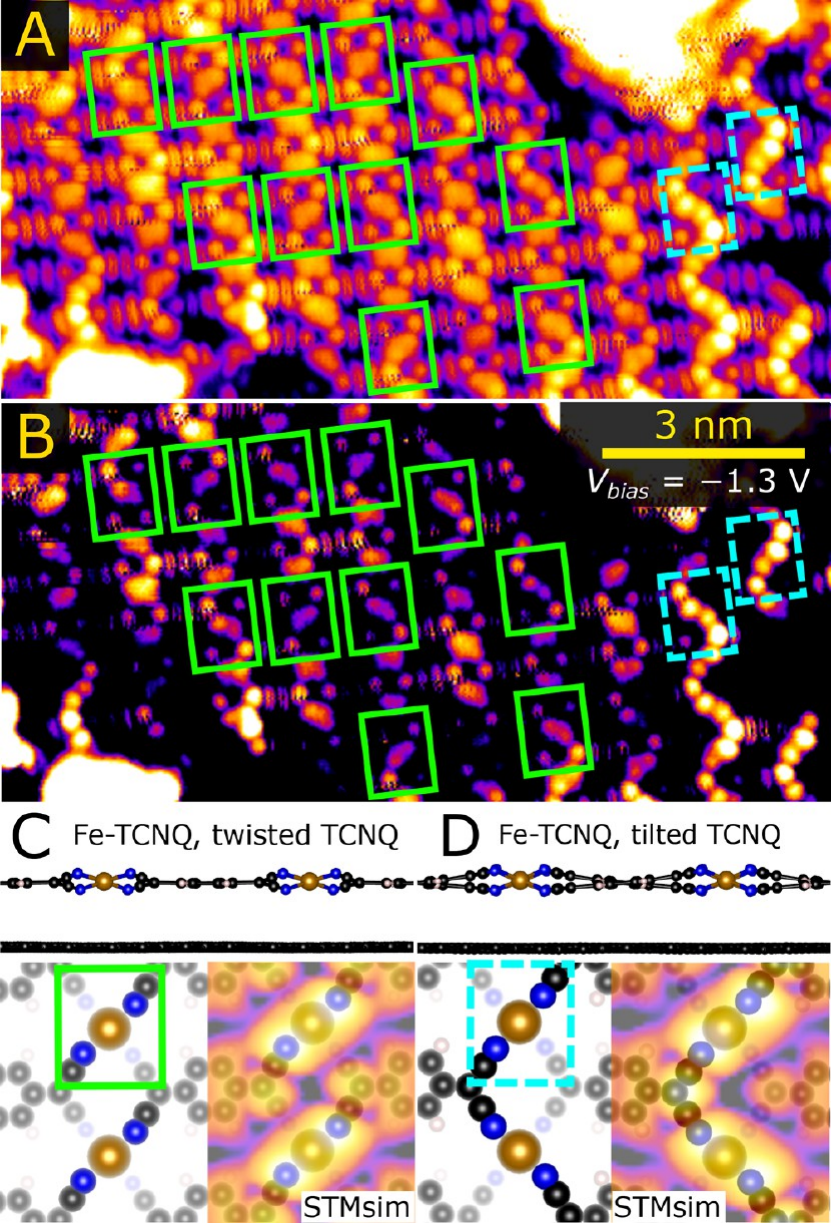
STM images of Fe-TCNQ/graphene taken at the areas that do not feature a distinct zigzag pattern. Panel A shows the original image, and panel B shows the same image with contrast adjustment highlighting that the Fe sites often look elongated (highlighted by green rectangles), qualitatively similar to the Fe sites in areas with the distinct zigzag (dashed cyan rectangles). We explain this local appearance by the model shown in panel C, where the quasi-tetrahedral Fe site is accommodated by twisting the TCNQ linkers. The height corrugation of this model is much lower than in the tilted-TCNQ model shown in panel D.

Having elucidated the origin of the zigzag pattern, we focus on the areas of the Fe-TCNQ/graphene that lack the zigzag appearance (Figure 2A). In Figure 2B, the contrast is enhanced to highlight that the local environment of Fe atoms (green rectangles in Figure 2A,B) often shows an elongated shape. This is similar to the Fe sites within the zigzag areas (dashed cyan rectangles), albeit less pronounced and lacking the long-range order. We interpret this appearance by an alternative structure of Fe-TCNQ 2D MOF, in which the quasi-tetrahedral Fe site geometry is accommodated by twisting the cyano groups of the TCNQ linkers (Figure 2C). DFT results show that on graphene, this structure is energetically comparable (within 20 meV per Fe_1_(TCNQ)_1_ unit) to the structure with tilted TCNQ linkers (Figure 2D). Further in this paper, we refer to these two models as “twisted-TCNQ” (Figure 2C) and “tilted-TCNQ” (Figure 2D). The height corrugation of the twisted-TCNQ structure is lower than in the tilted-TCNQ case (0.9 vs 1.2 Å on graphene), which explains its lower visibility in the STM images. This is also corroborated by STM simulations showing lower corrugation of the twisted-TCNQ phase (see insets in Figure 2C,D and Figure S5 in the SI). In experiments, the Fe-TCNQ layer often lacks long-range order and features a coexistence of the twisted- and tilted-TCNQ motifs. DFT computations confirm that such structures are slightly favored over ordered twisted-TCNQ phase but are still less favorable than the ordered tilted-TCNQ model (for details, see Figures S6 and S7 in the SI).

To evaluate how the Fe-TCNQ structure on graphene can be affected by the presence of the Ir support and corrugation of the graphene/Ir moiré, we performed DFT computations of various Fe-TCNQ models on planar and corrugated graphene sheets with and without the presence of the Ir(111) support. The results are presented in Section 4 of the SI and indicate that all the stability trends are the same as on the free-standing planar graphene sheet.

Thus, based on the STM/DFT data, we conclude that the Fe-TCNQ is nonplanar when supported on graphene and that the nonplanarity is most likely driven by the preference of the Fe center to reside in a tetrahedral environment. We note that twisted-TCNQ models were previously proposed for some metal-TCNQ systems,^28,29^ but the tilted-TCNQ structure was not reported.

**Figure 3:**
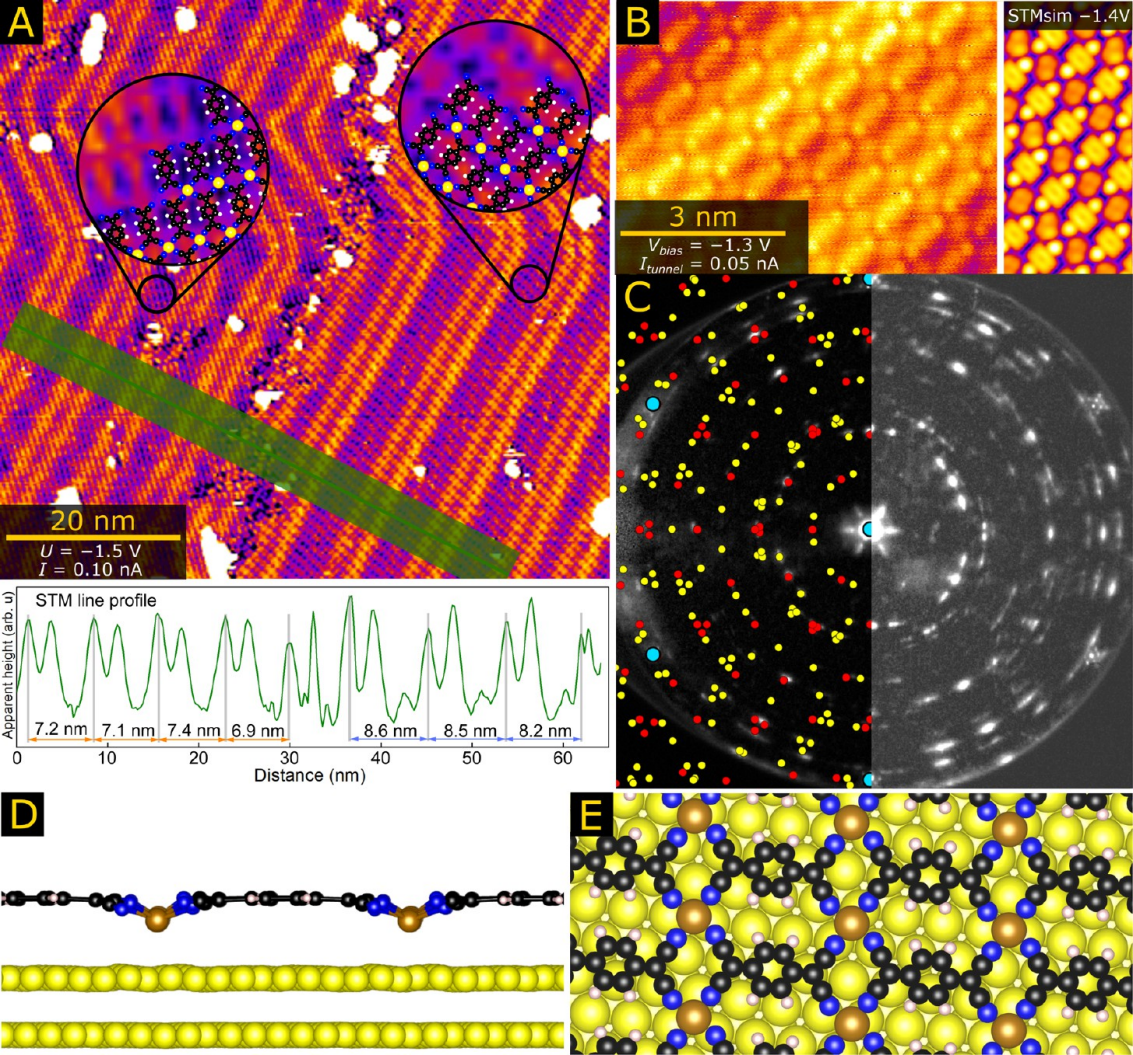
Fe-TCNQ supported on Au(111). (A) Large-scale STM images indicate a significant interaction between Fe-TCNQ and Au(111), which is manifested by the different periodicity of the herringbone reconstruction of the Au(111). Here, the image shows two patches of different Fe-TCNQ orientations (as shown in the zoomed-in insets), and the green line profile indicates that the herringbone periodicity changes by more than 20% under the two patches. (B) Local STM image shows well-ordered Fe-TCNQ on Au(111). The STM appearance is in good agreement with STM simulation (inset on the right) based on the planarized model shown in panels D and E. (C) LEED pattern originating from Fe-TCNQ/Au(111) can be well reproduced by two unique orientations of Fe-TCNQ on Au(111). The red spots correspond to the long axis of the Fe-TCNQ unit cell aligned with the Au(111), and the yellow spots correspond to Fe-TCNQ rotated by 13° from the Au(111). (D, E) The planarized DFT model of Fe-TCNQ/Au(111) with Fe atoms drawn closer to the Au(111) surface. This model provides better agreement to experimental data than tilted-TCNQ and twisted-TCNQ model (details in the SI).

The next important question is whether these structures featuring quasi-tetrahedral sites are only achievable on graphene or whether they could also be formed on commonly used metal supports. To address this, we grew excellent-quality Fe-TCNQ networks on Au(111) (Figure 3A,B) with domain sizes up to several hundred nanometers covering almost the entire Au(111) surface (mesoscale LEEM analysis shown in Figure S8). The details on the Fe-TCNQ synthesis are provided in the Methods section. The large domain sizes along with the presence of well-resolved Au(111) herringbone reconstruction under the Fe-TCNQ network tempt one to conclude that the network–substrate interaction is weak. However, a closer inspection of the line profile shown in the bottom panel of Figure 3A reveals that the spacing of the herringbone reconstruction varies by more than 20% under differently oriented Fe-TCNQ domains. This provides clear evidence that the presence of Fe-TCNQ changes the structure of the gold support. Analysis of low energy electron diffraction data (LEED, Figure 3C) and STM data (Figure S10) reveals that the nearest-neighbor distance in the top layer of Au(111) substrate is extended by up to several pm under the Fe-TCNQ, whereas the Fe-TCNQ unit cell may be slightly laterally compressed (details provided in Section 5 of the SI). In contrast to the graphene-supported case, the high-resolution STM images of Fe-TCNQ/Au do not show any hints of patterns formed by the tilting or twisting of TCNQ molecules (Figure 3B). Additionally, on Au(111), we locally observed several alternative Fe-TCNQ structures not found on graphene, a thorough analysis of which is beyond the scope of this paper (examples shown in Figure S10 in the SI).

We conducted DFT computations to elucidate the structure of the Au-supported Fe-TCNQ. We find that although both the tilted-TCNQ and twisted-TCNQ models can be stabilized on Au(111), we also identify an almost isoenergetic model featuring planarized TCNQ linkers with Fe atoms residing ≈1 Å closer to the support (Figure 3D). Further in this paper, we refer to this structure as “planarized”. Similar structures with metal atoms drawn to the support are common in metal-supported MOF systems.^19,26,30,31^ The planarized structure is stabilized by the van der Waals interaction between Fe and Au. This is clearly demonstrated by our test computations using the same setup without the vdW correction, in which the planarized model relaxes into the tilted-TCNQ structure. Thus, the structure of Fe-TCNQ 2D MOF on Au(111) is subjected to a competition between the preference of Fe to reside in tetrahedral geometry and the vdW interaction pulling the Fe toward the support. Within our computational setup (including vdW correction), all the tested models are energetically almost identical (the difference between the most and least stable model is 40 meV per Fe_1_(TCNQ)_1_). However, the planarized model provides the best agreement with the experiment, as shown in Figure 3B. The STM simulations of twisted- and tilted-TCNQ models look very different, as shown in Figure S12 in the SI. Additionally, the presence of long-range ordered tilted-TCNQ structures can be conclusively ruled out on the basis of LEED data (Figure 3C, more data in the SI).

Thus, we conclude that all of the tested structures can be locally stable on the Au(111) support, but experimental results indicate the prevalence of the planarized structure with Fe atoms drawn closer to the Au support. The stability of the planarized structure on Au(111) presents a major difference from that of the graphene-supported system, where a similar model does not present even a local energetic minimum.

**Figure 4:**
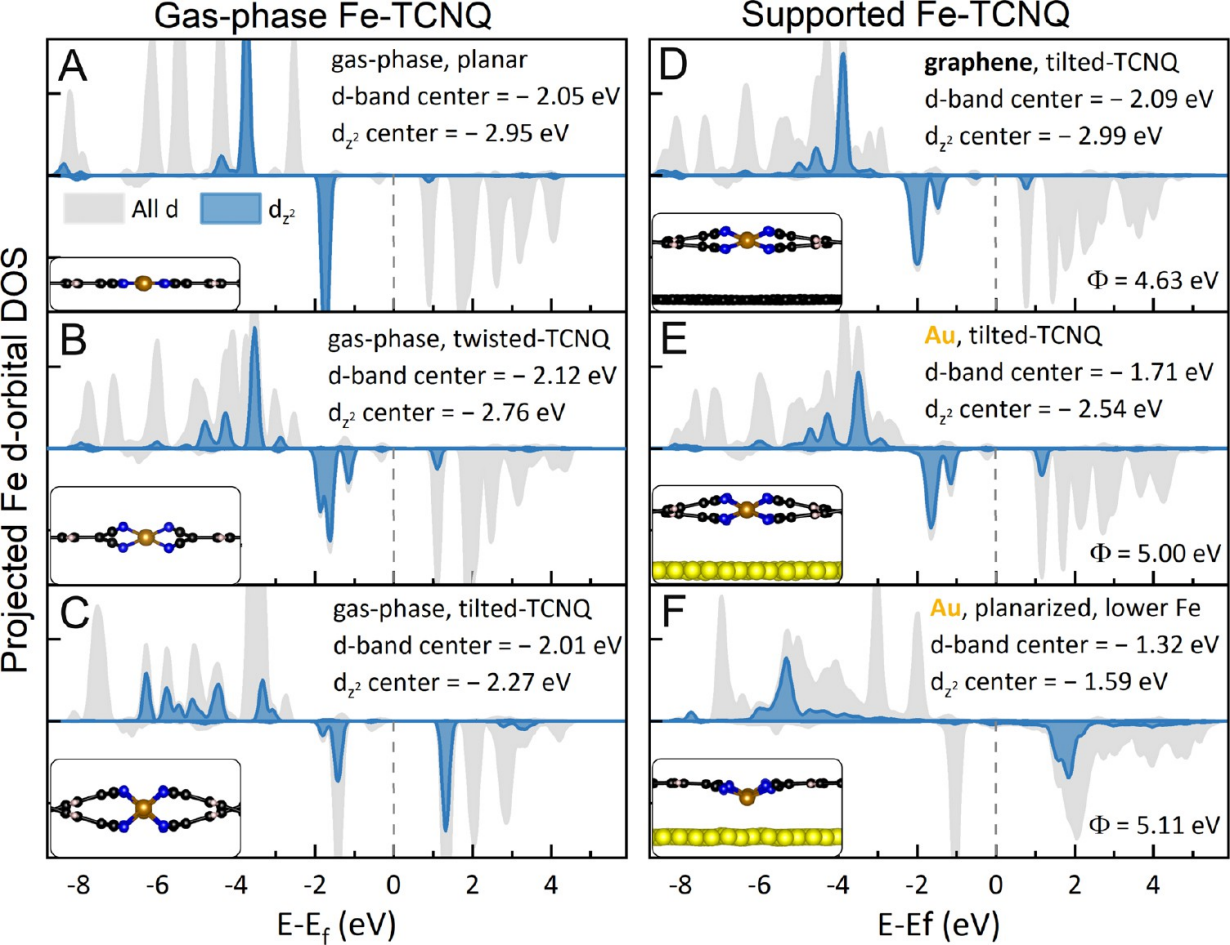
Density of states projected on the Fe atoms in the considered Fe-TCNQ models. The side view of the relevant structural model is shown in the bottom left corner of each panel. (A–C) Comparison of free-standing Fe-TCNQ models reveals the effects of the tetrahedral coordination on the Fe d-electron configuration. Notably, the occupancy and center of mass of the d_*z*^2^_ orbital are significantly changing between the planar, twisted-TCNQ, and tilted-TCNQ models. (D, E) On supported models, the electronic features related to tetrahedral coordination are much weaker because the quasi-tetrahedral Fe sites are slightly planarized. However, between tilted-TCNQ structures on graphene and Au(111), a significant 0.4 eV shift is observed as a result of the different work functions of the two supports. (F) In the planarized Fe-TCNQ structure, the effects of different d_*z*^2^_ occupancy and energy alignment with the support are combined, resulting in dramatically different positions of the d-band center, d_*z*^2^_ center, and d_*z*^2^_ filling in comparison to graphene-supported Fe-TCNQ. In panels D, E, and F, the work function values from DFT are given in the bottom right corner.

We analyzed the electronic and magnetic properties of the DFT models discussed in this paper to determine how the supports affect the properties of the supported 2D MOFs. With a view toward catalysis applications, we focused on the common descriptors of adsorption and catalytic activity, i.e., the filling of the d-orbitals and the positions of the d-band and d_*z*^2^_ orbital with respect to the Fermi level.^32−34^Figure 4 shows density of states (DOS) plots of the Fe d-orbitals in the individual models; the structures are shown in the inset of each panel. In the text, we follow the model names introduced earlier (gas-phase planar, twisted-TCNQ, tilted-TCNQ, and planarized on the Au(111) support). All the tested models are qualitatively similar in Fe charge state and magnetic moment, consistent with a Fe(II) d^6^ cation in an S = 2 quintet state (for details, see Table ST5 in the SI). However, we identify two parameters that define the d_*z*^2^_ filling and position of the d-orbitals with respect to the Fermi level: the local geometry of the Fe cation and the energy level alignment with the support.

The first parameter is structural, best illustrated by the *gas-phase* models shown in Figure 4A–C. As the Fe site geometry changes from square-planar (planar model, Figure 4A) to quasi-tetrahedral (twisted-TCNQ, Figure 4B) and tetrahedral (tilted-TCNQ, Figure 4C), the initially fully occupied d_*z*^2^_ orbital hybridizes with d-orbitals in the *x–y* plane. This results in the appearance of an unoccupied d_*z*^2^_ state at 1.3 eV, which is apparent in the twisted-TCNQ model (Figure 4B) and becomes dominant in the tilted-TCNQ model (Figure 4C, with additional details provided in the SI). The decreased filling of the d_*z*^2^_ orbital is associated with an upward shift of the d_*z*^2^_ center of mass by up to 0.7 eV.

Similar effects of the physical structure can also be observed on the *supported* Fe-TCNQ models. The DOS plots of tilted-TCNQ models supported on graphene (Figure 4D) and Au(111) (Figure 4E) look both very similar to the twisted-TCNQ model in the gas phase (Figure 4B). Specifically, we observe relatively sharp orbital states and only a small unoccupied d_*z*^2^_ state above the Fermi level. This can be rationalized by inspecting the Fe-site geometry (insets in Figure 4D,E), which is quasi-tetrahedral, similar to the gas-phase twisted-TCNQ model (inset in Figure 4B). Finally, we inspect the planarized Fe-TCNQ model with Fe atoms drawn to the Au(111) support (Figure 4F). Here, the orbital positions are significantly smeared, and we observe a dominant unoccupied d_*z*^2^_ state at 1.9 eV. Such behavior is not surprising because the Fe cation resides very close to the Au support; thus, the d_*z*^2^_ orbital spatially overlaps with the electron density from the Au support. To summarize, a qualitative interpretation of the structural influence on the d_*z*^2^_ center position is rather straightforward: in Fe-TCNQ, the d_*z*^2^_ is doubly occupied when it does not spatially overlap with the orbitals of the neighboring atoms. When it does partially overlap, either due to the significant nonplanarity of the MOF (Figure 4C) or due to the close vicinity to the support (Figure 4F), then the accommodation of two electrons in this orbital becomes unfavorable, and the d_*z*^2^_ filling is decreased. In Fe-TCNQ, such a d-electron rearrangement does not significantly change the charge state, magnetic moment, or d-band center position of the high-spin Fe(II) center, but it significantly reduces the occupancy of the d_*z*^2^_ orbital and shifts the position of the d_*z*^2^_ center.

The second parameter affecting the properties of supported 2D MOFs is energy level alignment with the support. This is best demonstrated on the models that are structurally almost identical but differ in the supporting surface, i.e., tilted-TCNQ on graphene and on Au(111). The DOS plots of these models show very similar features but are rigidly shifted by ≈0.4 eV (Figure 4D,E). This shift cannot be explained by the work-function difference between the clean supports alone, as that amounts to ≈0.7–0.9 eV.^35,36^ However, the support work functions are affected by the so-called push-back effect, which originates from the change in the electrostatic surface dipole induced by the presence of physisorbed adlayers.^37^ Our results show that this significantly affects the Au(111) support, the work function of which is reduced from 5.45 eV (clean surface) to 5.00 eV when the tilted-TCNQ is present. In the graphene-supported model, the push-back-induced change of the surface dipole is minimal because of the much lower electron density in the graphene support. Hence, the graphene work function remains almost unchanged (DFT indicates a slight increase from 4.56 to 4.64 eV). Overall, the difference between computed support work functions in the presence of the tilted-TCNQ structure is ≈0.4 eV, in perfect agreement with the observed shift in the DOS plots shown in Figure 4D,E. Because Fe-TCNQ has a band gap, this shift does not result in different charge transfer from the support. Nevertheless, systems with metallic characteristics (e.g., Ni-TCNQ^38^) would be affected, and different charge states and magnetic moments should be expected on these two supports even if the MOF structure is identical.

Finally, the most relevant comparison can be made between the structures that can be experimentally achieved, i.e., graphene-supported tilted-TCNQ (Figure 4D) and Au-supported planarized Fe-TCNQ with Fe atoms drawn to the support (Figure 4F). Between these two, the above-discussed effects are combined, resulting in vastly different positions of both the d-band center (by 0.8 eV) and d_*z*^2^_ center (by 1.4 eV). These differences are significant, and the specific implications for catalysis research are discussed below.

**Figure 5:**
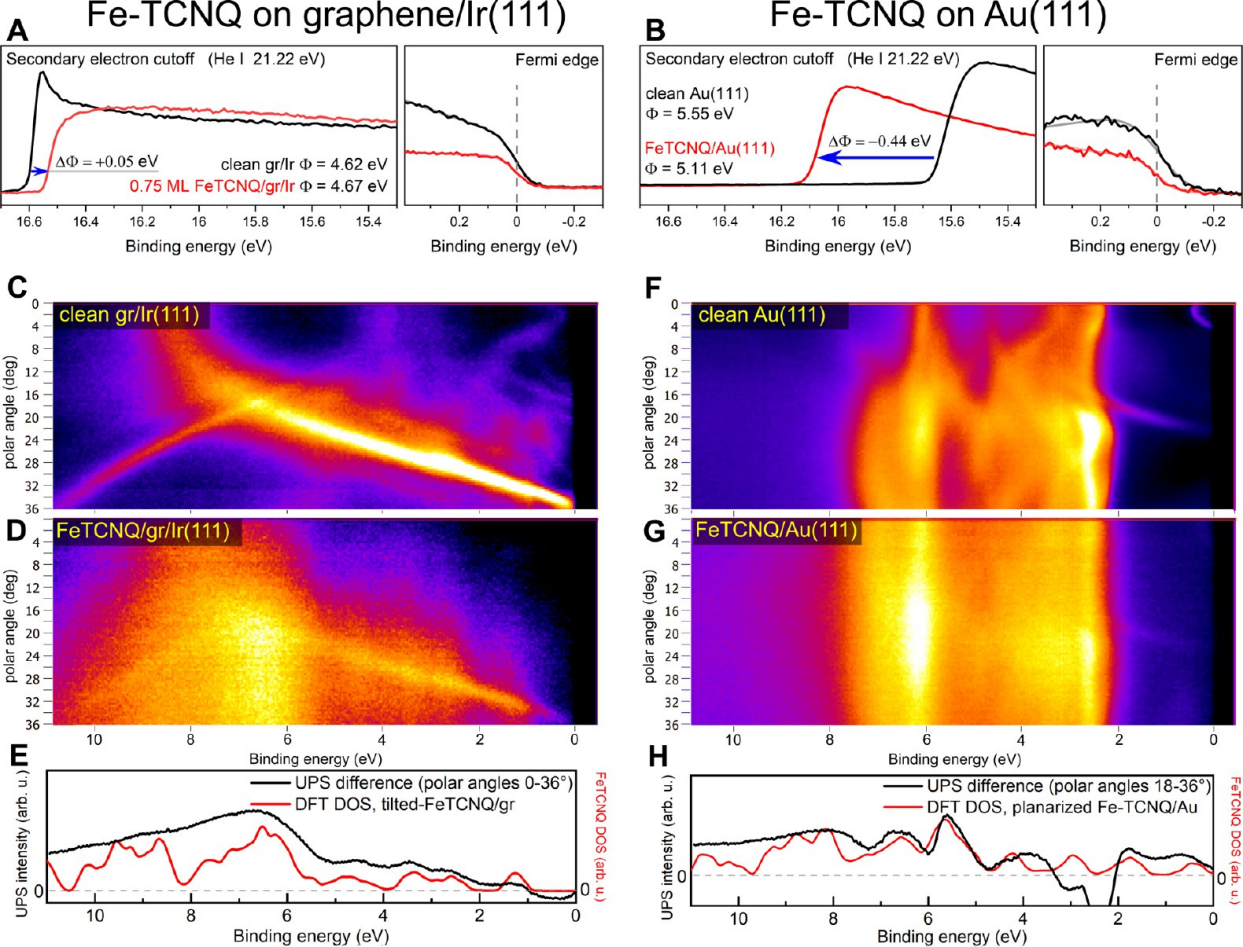
Ultraviolet photoemission spectroscopy (UPS) characterization of the Fe-TCNQ 2D MOFs on graphene and on Au(111). (A, B) Work function measurements acquired with He I (21.22 eV) excitation. (A) The work function of graphene is increased by 0.05 eV upon Fe-TCNQ deposition. (B) The work function of Au(111) is decreased by 0.44 eV upon Fe-TCNQ deposition. (C–H) Angle-resolved photoemission spectra acquired with He II (40.81) excitation. On graphene (C–E), a good agreement can be achieved between the experimental difference spectrum (black curve in panel E) and DFT data (red curve, see main text for details). (F–H) On Au(111), the agreement between the experiment and theory is good at higher binding energy but limited close to the Fermi level (H). The energy scale of the computational data was corrected as described in the text.

To corroborate the DFT results, we conducted ultraviolet photoemission spectroscopy (UPS) measurements. Figure 5A,B shows secondary electron cutoff positions measured with 21.22 eV (He I) excitation. Consistent with the computational results, the experiments show that the presence of Fe-TCNQ slightly increases the work function of graphene/Ir (from 4.62 to 4.67 eV with Fe-TCNQ coverage of ≈0.75 monolayer (ML)) but significantly decreases the work function of Au(111) (from 5.55 to 5.11 eV). The experimental work function measurements are thus in very good agreement with theory.

To gain further experimental insights into the electronic structure of the Fe-TCNQ 2D MOF, we measured angle-resolved photoemission spectra. Figure 5C shows the electronic structure of pristine graphene/Ir measured at polar angles between 0 and 36°; this data set is fully consistent with the literature.^39^ After deposition of ≈0.75 ML Fe-TCNQ (Figure 5D), the electronic structure of the graphene/Ir support is less discernible, and additional nondispersive bands are visible at ≈6.6 4.5, 3.5, 2.6, and 1.5 eV. Figure 5E shows a difference spectrum (black curve) calculated as the subtraction of spectra shown in panel C from panel D, normalized to low binding energy background, and averaged over the full polar angle range. To provide a direct comparison to the DFT data, we plot the computed Fe-TCNQ density of states in the same plot in red color. To correct for the well-known limitations of the DFT results obtained by generalized gradient approximation (GGA) functionals, the computational energy scale was extended by 30% and shifted down by 0.3 eV; such values are fully consistent with the literature.^40,41^ This correction shifts the absolute energy positions but does not affect the identified trends. Figure 5E shows that all the nondispersive bands related to Fe-TCNQ on graphene measured by UPS are well reproduced in the DFT data. Figure 5F,G shows a similar data set for the Fe-TCNQ/Au system. The difference spectrum averaged over polar angles 18–36° is shown in Figure 5H. The comparison to DFT data reveals reasonable agreement; most features observed in the experiment are reproduced by the planarized-TCNQ model. Additional DFT and UPS data are presented in the SI; we note that the differences in the total density of states between the individual computed Fe-TCNQ models are rather subtle. In any case, this data set shows that our DFT results are fully consistent with experiment.

The identified differences in d-orbital energy positions have strong consequences for potential applications. In catalysis, the adsorption energies of reactants on bulk metals or nanoparticles are typically correlated to the position and filling of the metal d-band. In single-atom catalysis, the position of the d_*z*^2^_ orbital was predicted to be a more accurate descriptor for adsorption strengths of upright-standing molecules.^32,33^ Our results indicate that the structural effects can shift d_*z*^2^_ by 0.2–0.9 eV, whereas an additional 0.4–0.5 eV shift comes from the work function difference between the two tested substrates. Because of our GGA-based computational approach, these are conservative estimates; the comparison to UPS data suggests that the d_*z*^2^_ shifts caused by structural effects can be up to 30% higher. Recent computational literature shows that such changes in d_*z*^2^_ position can result in huge differences in adsorption energies, even above 1 eV for some reactants.^32^ Such computational predictions are difficult to confirm on real working catalysts, but they can be efficiently tested and benchmarked by surface science experiments. Indeed, recent experimental reports show that the subtle interplay of structural relaxation and charge redistribution between the metal d-orbitals can be correlated to a >100 K shift in experimental CO desorption temperature from single-atom sites in almost identical local geometry.^42,43^

**Figure 6:**
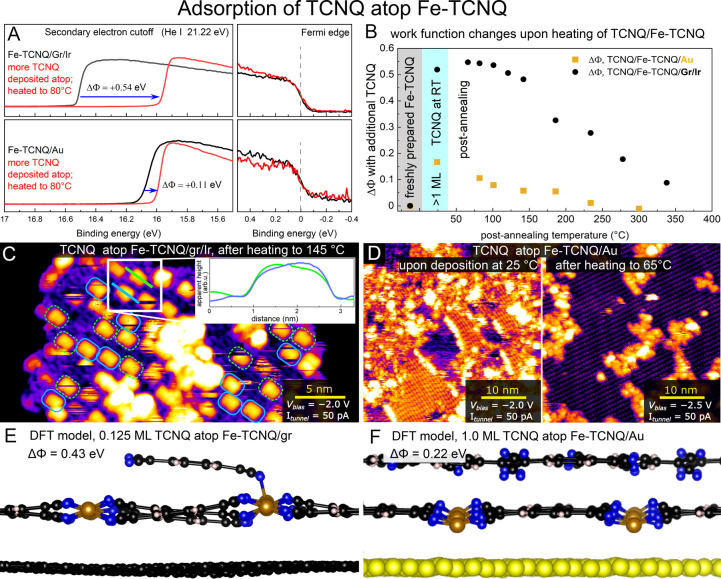
Multitechnique adsorption study of Fe-TCNQ using TCNQ as a probe molecule. (A) Work function measurements reveal that adsorption of TCNQ induces more than 0.5 eV work function increase on Fe-TCNQ/gr/Ir, whereas the same treatment induces only 0.1 eV increase of the Fe-TCNQ/Au work function. (B) The thermal stability of the work function increase shows that the work function of Fe-TCNQ/Au is recovered at 230 °C, whereas the work function of Fe-TCNQ/gr/Ir is still significantly increased at 350 °C. (C) STM images of TCNQ atop Fe-TCNQ/gr/Ir show isolated molecules with an asymmetric appearance. As highlighted by the blue and green line profiles and rectangles, each TCNQ molecule has one end brighter than the other. This is consistent with the DFT model shown in panel E. (D) STM images of TCNQ atop Fe-TCNQ/Au show the presence of close-packed TCNQ structures after deposition at 25 °C, but these structures disappear after heating to 65 °C. (E) DFT model of isolated TCNQ molecule adsorption on Fe-TCNQ/gr/Ir shows chemical bonding of the adsorbate leading to tilted geometry observed in STM and significant work function increase already at low TCNQ coverage. (F) DFT models of TCNQ atop Fe-TCNQ/Au show the absence of chemical interaction with the adsorbate and much lower increase of work function.

We carried out adsorption studies to probe whether similarly striking differences in the reactivity of single-atom sites could be observed between Fe-TCNQ 2D MOFs on the two “weakly interacting” supports. We chose TCNQ as a probe molecule, which is stable atop Fe-TCNQ above room temperature, allowing us to conduct a detailed multitechnique study. On both Fe-TCNQ systems, we have deposited >1ML of TCNQ at 25 °C and post-annealed the samples to 80 °C to desorb any TCNQ multilayers^44^ and TCNQ molecules from areas of clean graphene/Ir.^27^ The UPS work function measurements shown in Figure 6A reveal that upon this treatment, the work function of Fe-TCNQ/gr/Ir increases by more than 0.5 eV, whereas the Fe-TCNQ/Au work function only increases by 0.1 eV. This clearly indicates that the electrostatic surface dipole changes differently on the two supports, hinting on different structures and/or bonding mechanisms taking place. We have also measured the thermal stability of the observed work function changes. As plotted in Figure 6B, the work function of Fe-TCNQ/gr/Ir remains increased by 0.5 eV up to 150 °C; above this temperature, it decreases as the TCNQ gradually desorbs. Still, even after heating to 350 °C, a work function difference of 0.1 eV is observed, indicating that some TCNQ molecules remain strongly bound to the Fe-TCNQ/gr/Ir system. In contrast, the work function increase of Fe-TCNQ/Au falls below 0.1 eV upon heating above 100 °C, and the original work function value is restored upon heating to 230 °C. The UPS data set thus indicates that the adsorbate bonding mechanisms are likely different, and the thermal stability of TCNQ atop Fe-TCNQ/gr/Ir is much higher than atop Fe-TCNQ/Au.

We carried out STM and DFT characterizations of the TCNQ/Fe-TCNQ systems to support the conclusions from UPS measurements. Figure 6C shows an STM image of Fe-TCNQ/gr/Ir after additional TCNQ deposition and heating to 145 °C. The image shows individual adsorbed TCNQ molecules as isolated bright species atop Fe-TCNQ. The appearance of adsorbed TCNQ is slightly asymmetric, as highlighted by the green and blue line profiles in the inset. This appearance is perfectly consistent with our DFT computations, which reveal that an isolated TCNQ molecule is chemically bound to two underlying Fe cations via two of its four −CN groups, leading to a slight tilt of the molecule. Our DFT computations indicate that the newly formed CN–Fe bonds are associated with a strong vertical charge transfer, which significantly increases the work function (Figure 6E). Already at a low TCNQ coverage of 0.125 ML, the computed work function is increased by 0.43 eV. This is consistent with the UPS data, where a work function increase of ≈0.3 eV is still detected even after heating above 200 °C (note that, in the experiment, the Fe-TCNQ coverage is only about 70%).

The STM images of the Au-supported TCNQ/Fe-TCNQ system are shown in Figure 6D. Directly after deposition at 25 °C (prior to post-annealing), the STM images show the presence of additional material, which looks disordered on parts of the image but also features some areas of ordered close-packed structures. However, already upon heating to ≈65 °C, these close-packed structures disappear, and the STM images show only areas of pristine Fe-TCNQ, with the adsorbed TCNQ being present mainly near domain boundaries and defects within the Fe-TCNQ layer (for details of this assignment, see Figure S17 in the SI). This is consistent with the UPS data, where the TCNQ-induced work function difference is minimal already upon mild heating. We designed several DFT models of a close-packed TCNQ monolayer atop Fe-TCNQ/Au to evaluate the adsorbate-induced work function changes (one model is shown in Figure 6F, others are presented in Figure S18 in the SI). Even though the models do not exactly capture the experimentally observed periodicity (which is too large for DFT computations), they all show similar properties, allowing us to estimate the main trends in TCNQ adsorption. Specifically, the interaction between the adsorbed TCNQ and underlying Fe-TCNQ/Au is mainly of vdW and electrostatic origin in all the tested models; i.e., none or very few chemical bonds are formed. A delocalized vertical charge transfer takes place nonetheless, most likely through π–π stacking. The work function of all of the tested models was only 0.22–0.32 eV higher than that of pristine Fe-TCNQ/Au despite the very high (1 ML) TCNQ coverage. These values are in good agreement with the 0.17 eV measured by UPS directly after deposition (where only a part of the surface is covered by the close-packed structure, as measured by STM).

Our multitechnique study of TCNQ adsorption thus confirms that the chemical reactivities of Fe-TCNQ atop graphene/Ir and Au(111) are dramatically different. On gr/Ir, the adsorbed TCNQ forms strong chemical bonds with the Fe-TCNQ support, whereas on Fe-TCNQ/Au, it is only weakly physisorbed. The reactivity of single-atom sites generally depends on the electronic structure as well as steric effects. For the case of large adsorbate molecules such as TCNQ, the steric effects likely dominate, which renders the lower-lying Fe cations in Au-supported Fe-TCNQ unreactive. However, for smaller upright-standing molecules, the electronic effects may prevail, and the reactivity trend might be completely the opposite, as recently observed in Ru-porphyrin systems.^42,43^ Overall, our work clearly shows that the chemical reactivity of 2D MOFs atop “weakly-interacting” supports can greatly differ, which clearly outlines the limits of common on-surface synthesis approaches using metal supports for the modeling of technologically relevant catalytic processes. On a more positive note, our work also demonstrates that graphene-supported 2D MOFs possess similar properties as their free-standing counterparts and can thus serve as perfect experimental models of the applied systems.

The research of metal-TCNQ 2D MOFs is directly relevant for applications, as these systems are promising single-atom catalysts for many reactions, including CO_2_ reduction or oxygen evolution.^12,16,45−47^ However, the relevance of our results is not limited to metal-TCNQ and can be broadly applied to all supported systems, including models for the highly promising single-atom catalysts based on N-doped graphene.^48−50^ We propose that graphene-supported metal-TCNQ systems can serve as perfect models for such catalysts, as they feature the same local environment of the metal (coordinated to four nitrogen atoms, metal-N_4_), they utilize a similar supporting material (graphene), and they can be studied with atomic-scale precision using on-surface approaches. Moreover, the performance of single-atom catalysts is undoubtedly affected by local doping or charge-transfer effects, and the dopable graphene support offers a unique opportunity to experimentally model such effects. The protocols of graphene doping by electric field or heteroatom intercalation are well-established, and graphene-supported 2D MOFs can thus become ideal test beds for fundamental research of single-atom reactivity.

## Conclusions

In summary, we have studied the properties of Fe-TCNQ 2D metal–organic frameworks on two weakly interacting supports: Au(111) and graphene/Ir(111). By a combined experimental and computational approach, we have conclusively shown that Fe-TCNQ on graphene is nonplanar with the iron atoms residing in quasi-tetrahedral sites. On Au(111), the experimental results suggest a slight lateral compression of the Fe-TCNQ layer, whereas DFT indicates that the Fe atoms are drawn closer to the Au support. The significant structural differences and the distinct work functions of the two supports dramatically change the chemical reactivity of the 2D MOF. A difference of 1.4 eV is observed between the Fe d_*z*^2^_ positions in Fe-TCNQ on the two supports, which markedly changes the predicted adsorption strengths on the Fe–N_4_ single-atom sites within the 2D MOF. Using a TCNQ probe molecule, we have shown that this large adsorbate molecule forms strong chemical bonds with Fe-TCNQ on graphene/Ir, whereas it only physisorbs on Fe-TCNQ/Au. This is most likely due to steric hindrance effects of the sunk-down Fe site on the Au(111) support. Overall, our data clearly indicate that despite the qualitative similarities of the 2D MOFs on the two weakly interacting supports, they must be expected to behave very differently in applied systems. The graphene-supported 2D MOFs partially retain the intrinsic properties and can serve as perfect experimental models for single-atom catalysis research.

## Methods

Experiments were carried out in an ultrahigh vacuum system consisting of multiple chambers interconnected by a central transfer line separated by gate valves. The base pressure of all the chambers used in this study is below 5 × 10^–10^ mbar. The Ir(111) single crystals (supplied by MaTecK and SPL) were cleaned by cycles of Ar^+^ sputtering (1.8 keV, 10 min) and flashing up to 1350 °C followed by annealing to 1080 °C (10 min). When graphene was present on the sample prior to cleaning, the first annealing cycle took place in O_2_ background (1160 °C, *p*_O_2__ = 1 × 10^–6^ mbar). The Au(111) crystals were cleaned by Ar^+^ sputtering and annealing to 540 °C. The temperature was measured with a LumaSense IMPAC IGA 140 pyrometer with the emissivity set to 0.1.

Graphene on Ir(111) was grown by adsorbing saturation coverage of ethylene at room temperature followed by pumping out the ethylene background and ramping the temperature up to 1250 °C in UHV. Then, the sample was re-exposed to ethylene at this temperature (1 × 10^–6^ mbar, 5 min). This protocol combines temperature-programmed growth (TPG) with chemical vapor deposition (CVD)^51,52^ and consistently leads to a full monolayer coverage of high-quality graphene/Ir(111).

For the Fe-TCNQ synthesis, TCNQ was thermally evaporated from a quartz crucible heated to 115 °C (MBE Komponenten OEZ), and iron was evaporated from an effusion cell (MBE Komponenten HTEZ). The evaporation rate of metals was checked by a water-cooled quartz crystal microbalance. The temperature during Fe-TCNQ synthesis was calibrated by a special sample holder with a K-type thermocouple attached close to the crystal surface. The Fe-TCNQ synthesis protocol involved the saturation of the substrate by TCNQ at a temperature close to a TCNQ desorption temperature followed by the codeposition of Fe and TCNQ at the same temperature. The TCNQ desorption temperature was determined by XPS measurements and was found to be ≈80 °C on graphene/Ir(111) and ≈140 °C on Au(111). After the codeposition, the samples were annealed to 340 °C, unless stated otherwise in the text. A detailed experimental analysis of graphene-supported metal-TCNQ systems is provided in ref (27).

Scanning tunneling microscopy images were recorded at room temperature in constant-current mode using a commercial system (Aarhus 150, SPECS) equipped with a Kolibri Sensor using a tungsten tip. Distortion in the STM images was corrected to fit the known dimensions of graphene/Ir moiré unit cell or spacing of the pristine Au(111) herringbone reconstruction. Where possible, nonlinear image distortion was corrected as described in ref (53). Low energy electron microscopy/diffraction experiments were carried out in a SPECS FE-LEEM P90 instrument. LEED patterns were simulated by LEEDpat 4.2.^54^ Ultraviolet photoemission measurements were carried out at room temperature by using a high-intensity SPECS UVS 300 UV source and a SPECS Phoibos 150 analyzer with a 2D detector. For the work function measurements, the sample was biased by −6 V.

All calculations based on the spin-polarized density functional theory (DFT) were performed with the Vienna *ab initio* Simulation Package (VASP)^55^ using the projector augmented wave method (PAW)^56^ to treat core electrons. We used a nonlocal van der Waals corrected optPBE-vdW functional^57^ for the description of exchange correlation energy. A Hubbard-like coulomb repulsion correction *U*-*J* = 4 eV in Dudarev’s formulation^58^ was considered for an appropriate description of Fe 3d orbitals. Different values of the Hubbard term *U*-*J* (between 3 and 6 eV) were tested on FeTCNQ in the gas phase and on the Au(111) support, and the marginal differences in the results do not affect the conclusions of this study. The robustness of the results was also tested by comparison to another class of vdW scheme, a Grimme’s atom-pairwise dispersion correction^59^ to the PBE functional.^60^ For iron, nitrogen, hydrogen, and carbon, 16 valence electrons (3s^2^3p^6^4s^2^3d^6^), 5 valence electrons (2s^2^2p^3^), 1 valence electron (1s^1^), and 4 valence electrons (2s^2^2p^2^), respectively, were expanded in a plane-wave basis set with an energy cutoff set to 520 eV. The Brillouin zone was sampled with a Γ-centered Monkhorst–Pack grid^61^ using more than 18 k-points per Å^–1^ for all the structural relaxations and 28 k-points per Å^–1^ for the subsequent electronic structure calculations. Structural optimizations were stopped when all residual forces acting on atoms in a system were less than 0.02 eV/Å.

For interface calculations, a four Au(111) layer thick slab and a single graphene sheet were considered as a substrate. The effect of the underlying Ir(111) surface present under graphene was also computationally tested, as described in the SI. To create the interface models, we kept the substrate atoms at their positions calculated from DFT, adjusted the molecular layer accordingly, and added a 15 Å thick vacuum layer. Molecular layers consisting of four Fe atoms and four TCNQ molecules were found to be a good compromise between the stress induced by a fit to the supercell and the orientation of the molecular layer with respect to the substrate on the one hand and the overall size of the system on the other hand. Further information on the models used is provided in the Supporting Information.

## Data Availability

The data underlying this study are available in the published article and its Supporting Information. The primary experimental data sets and used computational models are openly available in the Zenodo repository at 10.5281/zenodo.10454314.

## Supporting Information Available

Additional experimental and computational data; details about the computational unit cells (PDF)
LEED patterns of Fe-TCNQ/Au(111) at electron energies between 5 and 40 eV (the energy step between frames is 0.5 eV) (AVI)

## Supplementary Material

ja3c13212_si_001.pdf

ja3c13212_si_002.avi
