# Supplementary material for: How the Support Defines Properties of 2D Metal–Organic Frameworks: Fe-TCNQ on Graphene versus Au(111)
Source: J Am Chem Soc. 2024 Jan 22;146(5):3471–82. doi: 10.1021/jacs.3c13212 (PMC10859937; doi:10.1021/jacs.3c13212)
Supplement: Supplementary file 1 — ja3c13212_si_001.pdf [file ja3c13212_si_001.pdf]

Supporting Information

How the support defines properties of 2D metal-organic frameworks: Fe-TCNQ on graphene *versus* Au(111)

Zdeněk Jakub[1*], Azin Shahsavar[1], Jakub Planer[1], Dominik Hrůza,[1] Ondrej Herich[1], Pavel Procházka[1], Jan Čechal[1,2]

[1] CEITEC – Central European Institute of Technology, Brno University of Technology, Purkyňova 123, 61200 Brno, Czech Republic

[2] Institute of Physical Engineering, Faculty of Mechanical Engineering, Brno University of Technology, Technická 2896/2, Brno, Czech Republic.

Contents

1. Additional STM data of Fe-TCNQ/graphene 2
2. Considered supercell for Fe-TCNQ/graphene, comparison to LEED data 4
3. STM simulations of different Fe-TCNQ/graphene models 5
4. The effect of the underlying Ir(111) support and the resulting moiré pattern 5
5. Additional experimental data on Fe-TCNQ/Au 7
6. Considered supercell for Fe-TCNQ/Au(111), comparison to experimental data 10
7. STM simulations of different Fe-TCNQ/Au models 11
8. Fe-TCNQ lattice parameters from experiment and theory 12
9. Stability of Fe-TCNQ in the gas phase 12
10. Stability of Fe-TCNQ molecular layer on graphene and on gold 12
11. The energy penalty associated with designing commensurate structure models 13
12. The effect of lateral strain on the electronic structure of planarized FeTCNQ/Au 14
13. Comparison of the two models for gas-phase tilted-TCNQ structure 15
14. Spin-polarization of all the considered models 15
15. Comparison of the individual models to the UPS spectra 15
16. Polar angle dependence of the UPS difference spectra 16
17. Additional STM images of Fe-TCNQ/Au before and after TCNQ adsorption 16
18. DFT models of monolayer TCNQ atop Fe-TCNQ/Au 17
19. Description of Supplemental movie 1 18

References 18

1. Additional STM data of Fe-TCNQ/graphene

Figures S1-S3 provide additional STM images of graphene-supported Fe-TCNQ. The pronounced zig-zag pattern is clearly observed in different orientations, providing evidence that the zig-zag appearance is not just an artifact caused by an asymmetric STM tip. In Figure S3, the symmetry axes of the individual Fe-sites are highlighted, giving some idea of the long-range ordering of the tilted-TCNQ and twisted-TCNQ phases.

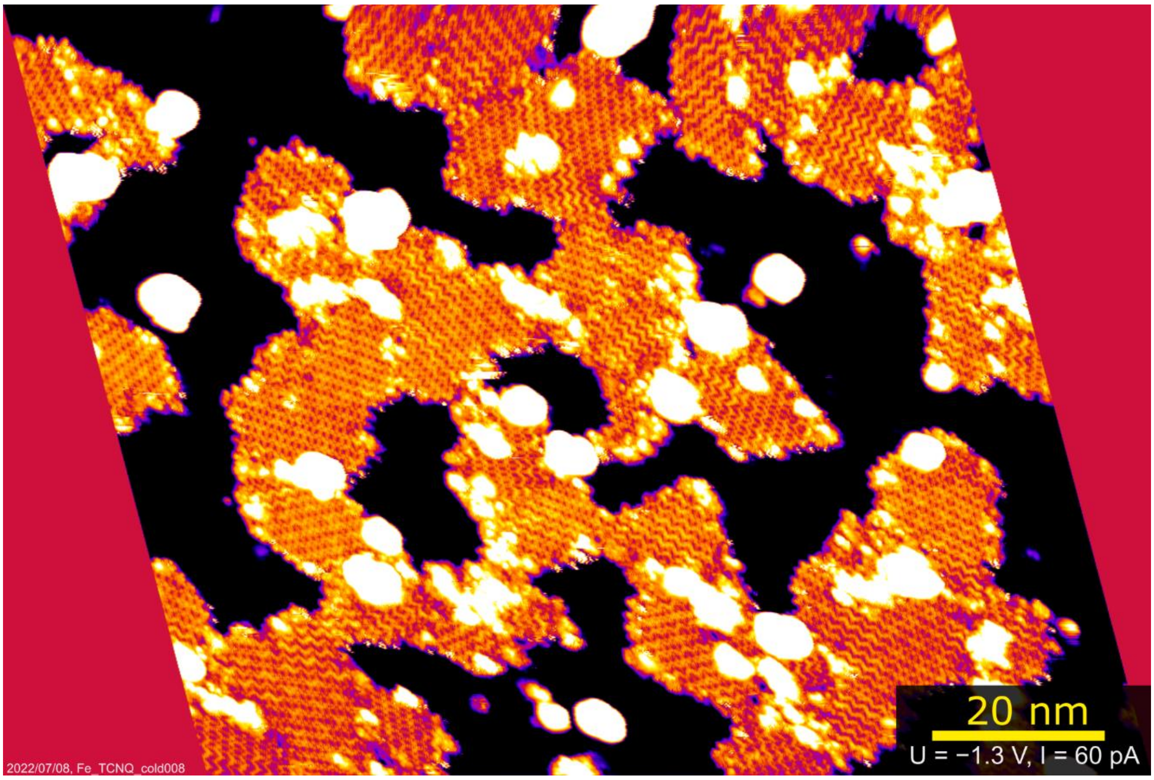

Figure S1: A large-scale STM image of graphene-supported Fe-TCNQ following the preparation described in the main text. The image was taken at 150 K, and features a significant fraction of the distinct zig-zag appearance. The zig-zag pattern is clearly resolved in many different orientations.

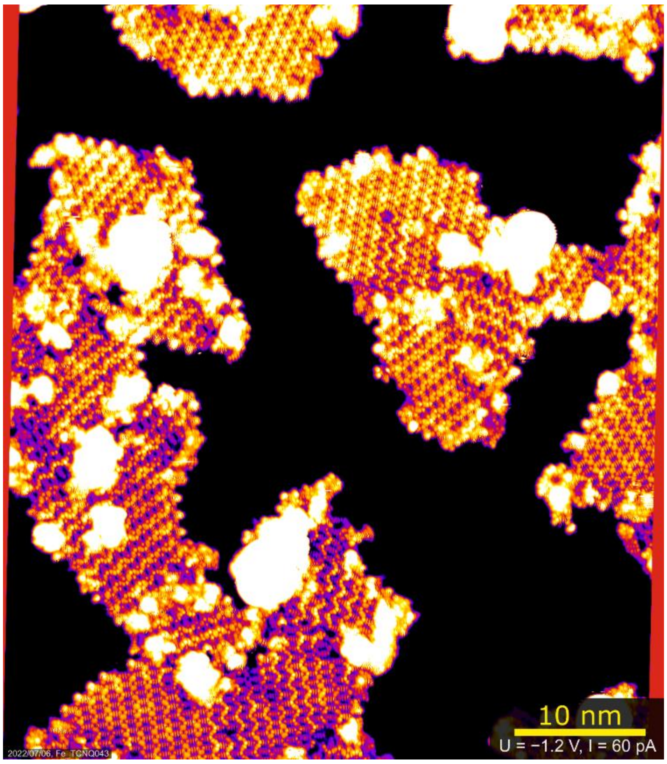

Figure S2: A room temperature, large-scale STM image of the graphene-supported Fe-TCNQ network.

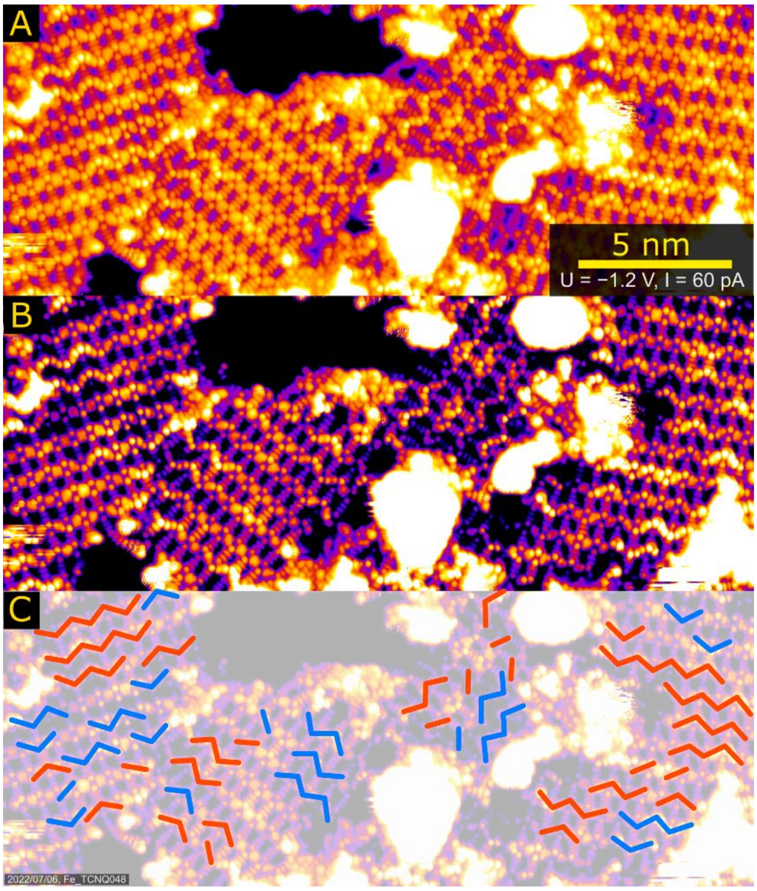

Figure S3: A detailed room-temperature STM image of a graphene-supported Fe-TCNQ network. Panel A shows the original image, panel B is contrast-adjusted for better visibility of the Fe-site shapes, panel C highlights the elongation of the Fe-sites. This provides some idea of how the tilted-TCNQ and twisted-TCNQ features are distributed in the experimental system.

2. Considered supercell for Fe-TCNQ/graphene, comparison to LEED data

Figure S4 shows the top view on the supercell of graphene-supported Fe-TCNQ. We found a reasonable match between the graphene supercell with the matrix notation $\begin{pmatrix} -4 & -13 \\ 5 & 1 \end{pmatrix}$ and the FeTCNQ layer with the matrix notation $\begin{pmatrix} 4 & 0 \\ 0 & 1 \end{pmatrix}$. This graphene-supported FeTCNQ unit cell is slightly compressed with respect to the gas-phase z-frozen planar model, but slightly extended with respect to the tilted-TCNQ gas phase model. Specifically, the match of the substrate and the planar gas-phase FeTCNQ molecular layer yields small tensile strain compared to the gas-phase tilted-TCNQ model by 1.4% in a direction, 5.8% b direction, and 1.8% angular strain. With respect to the z-frozen planar model in gas phase the strain is compressive, by -2.5% in a direction, -1.0% b direction, 1.8% angular. The dimensions of all the considered models are listed in Table ST1 below. The FeTCNQ orientation in the computational model agrees with the most common orientation found in experiment (panels B-D and reference[1]). Interestingly, some contrast-adjusted LEED patterns sometimes (Figure S4D) show slightly increased intensity in positions where the diffraction spots related to tilted-TCNQ phase might be expected. This may suggest existence of bigger tilted-TCNQ patches somewhere on the sample, with the long-range order better than the few-nm that we found in STM. Nevertheless, this interpretation is only tentative as the signal intensity is close to the noise level.

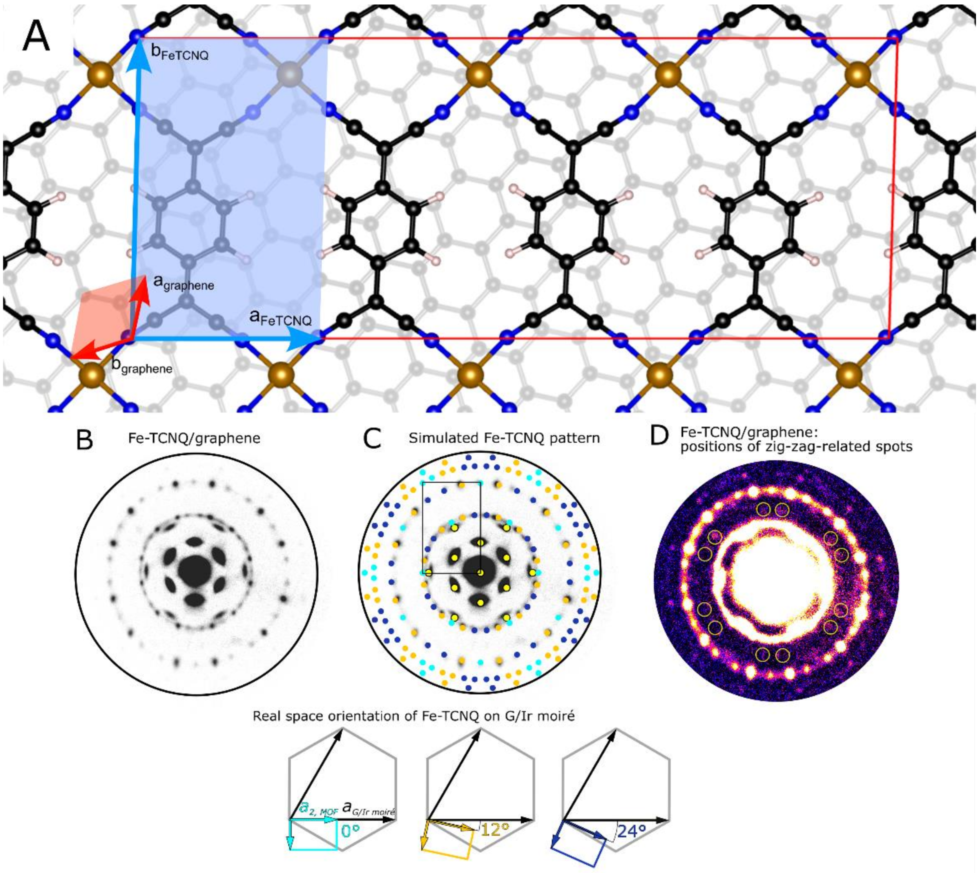

Figure S4: (A) Computational unit cell used for DFT simulations for Fe-TCNQ/graphene. The unit cell contains four Fe atoms, and the longer axis of the Fe-TCNQ is rotated by ca. 11° from the graphene unit cell, which agrees with the most intense spots seen in LEED (panel B, orange spots in panel C). The LEED primary electron energy was 16 eV. Panel (D) highlights the positions of potential diffraction spots related to the zig-zag pattern originating from tilted-TCNQ areas. Pronounced spots are not expected due to the very small areas and low coverage of the ordered tilted-TCNQ phase observed in STM (characteristic dimensions max. 5-10 nm). Nevertheless, the processed LEED pattern shows slightly increased intensity in some positions which would be consistent with this tilted-TCNQ phase.

3. STM simulations of different Fe-TCNQ/graphene models

Figure S5 shows STM simulations at a sample bias -1.6 V of the z-frozen planar model, twisted-TCNQ model and tilted-TCNQ model.

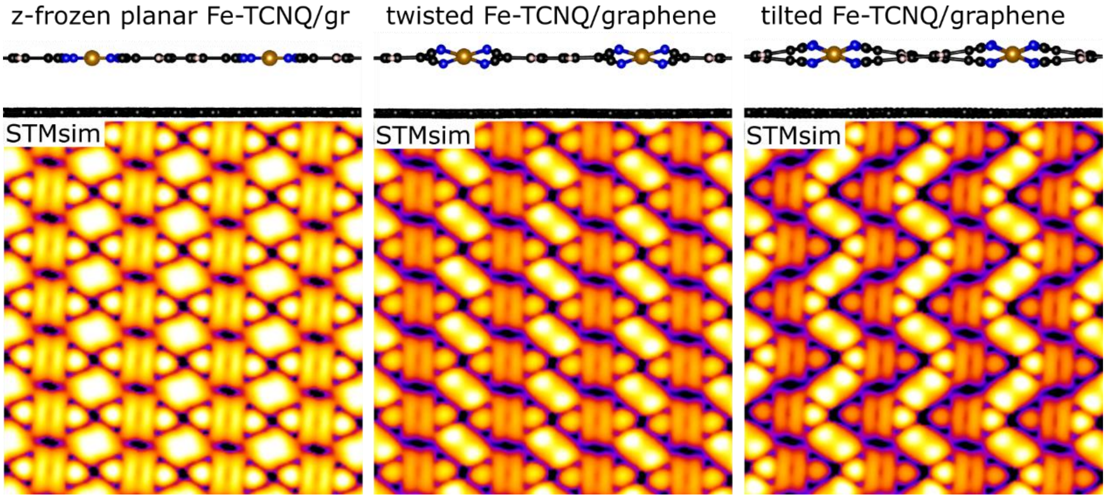

Figure S5: STM simulations of the different models considered for Fe-TCNQ on graphene.

4. The effect of the underlying Ir(111) support and the resulting moiré pattern

In the experimental graphene/Ir system, the lattice mismatch between the graphene and iridium results in a characteristic moiré pattern. Within the moiré unit cell, the graphene is physically corrugated by ≈ 0.4 Å, and its work function varies by ≈ 0.1 eV.[2,3] Both the physical and electronic corrugations can in principle affect the 2D MOF synthesized atop. Here we provide additional data and discussion which indicate that our main conclusions are not affected by the graphene/Ir moiré.

The corrugation of the work function can lead to site-specific differences in support-MOF charge transfer. However, all the tested Fe-TCNQ models only have minimal density of states near the Fermi level (see Figures 1, 4 and 5 in the main text), thus shifts in ≈ 0.1 eV range do not significantly change the charge distribution at the graphene/MOF interface.

The physical corrugation of the graphene/Ir moiré can affect the structure of the Fe-TCNQ 2D MOF. To test the possible extent of this effect, we designed two different models of corrugated graphene sheets, which we consequently use as fixed supports for Fe-TCNQ. The first model was formed by relaxing a planar graphene sheet atop an Ir(111) slab of 4 layers. To form a commensurate structure, the Ir slab is under slight lateral strain (+1.5 % in a, -1.6% in b). After relaxation, this results in a graphene/Ir moiré unit cell with a graphene height corrugation of ≈ 0.3 Å and lateral dimensions $a = 30.3$ Å$, b = 22.6$ Å, $\theta = 110°$ (see Figure S6). The second model of corrugated graphene was designed by modulating the height of the carbon atoms by a function $h = 0.27 * \cos[(x + y) * \pi] * \cos[(x - y) * \pi]$ across a 2 x 1 cell used previously for calculations of Fe-TCNQ/planar graphene (x and y are fractional coordinates of the carbon atoms, h is height in Å). This height modulation results in graphene corrugation of 0.54 Å across a unit cell with lateral dimensions $a = 28.4$ Å, $\mathrm{b} = 22.6$ Å, $\theta = 91.6°$ (see Figure S7). While both these models differ from the most common graphene/Ir moiré unit cell ($a = b = 25.3$ Å, $\theta = 120°$, height corrugation ≈ 0.4 Å), they are suitable for testing the effects of the support corrugation on the structure of 2D MOFs atop. We relaxed different Fe-TCNQ models supported on these corrugated graphene sheets while keeping the graphene fixed. The results are very similar to the conclusions from planar graphene models, as summarized in Figure S6 and S7.

First, the Fe-TCNQ with square-planar Fe sites is unstable. Tilted-TCNQ model is the most stable structure, followed by a model featuring both tilted-TCNQ and twisted-TCNQ motifs (which originated from relaxing a planar structure). Ordered twisted-TCNQ model is slightly disfavored, but the energy differences between all the non-planar structures are marginal, within 15 meV per $Fe_1(TCNQ)_1$ unit.

Based on these computational results we conclude that the graphene height corrugation in the range of 0.3-0.5 Å with lateral spacing between 22-30 Å does not affect the main conclusions of our study.

Lastly, we tested whether the results can be affected by the presence of the Ir(111) support in our computational model. We included the Ir(111) support back into the model shown in Figure S6, and we calculated the forces acting on the atoms of the Fe-TCNQ structure. All these forces remained below the convergence criterion of 0.02 eV/Å. We can thus safely conclude that the presence of Ir(111) below a corrugated graphene sheet does not change our results in any way.

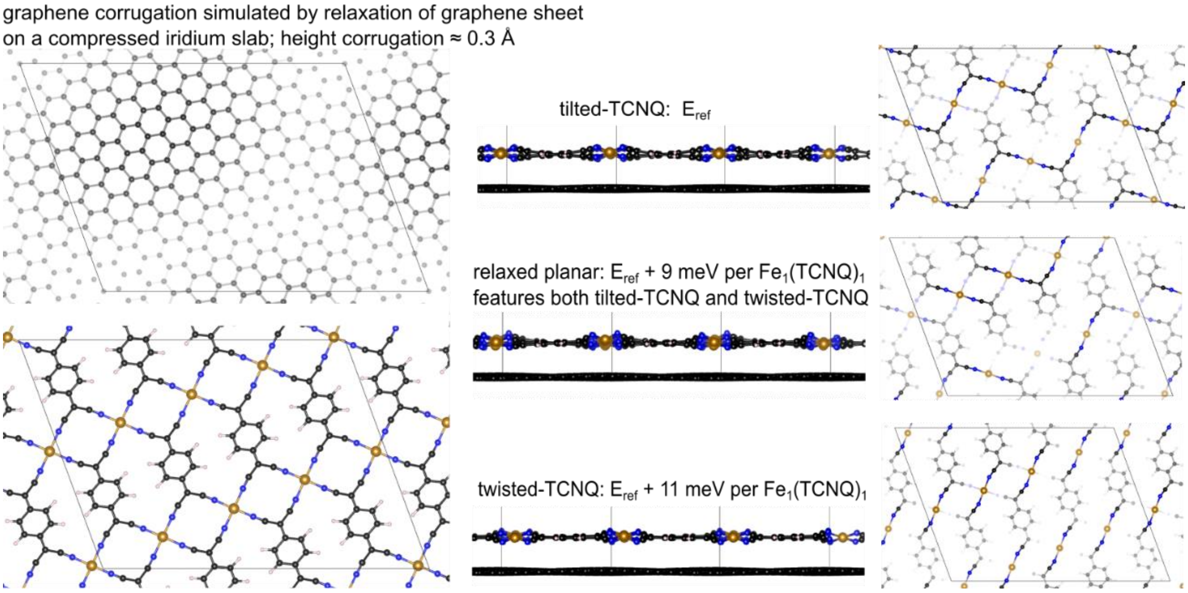

Figure S6: The computational unit cell of corrugated graphene sheet designed by relaxation of graphene atop slightly strained iridium slab. The Fe-TCNQ models relaxed atop this corrugated graphene sheet show the same stability trends as on planar graphene sheet.

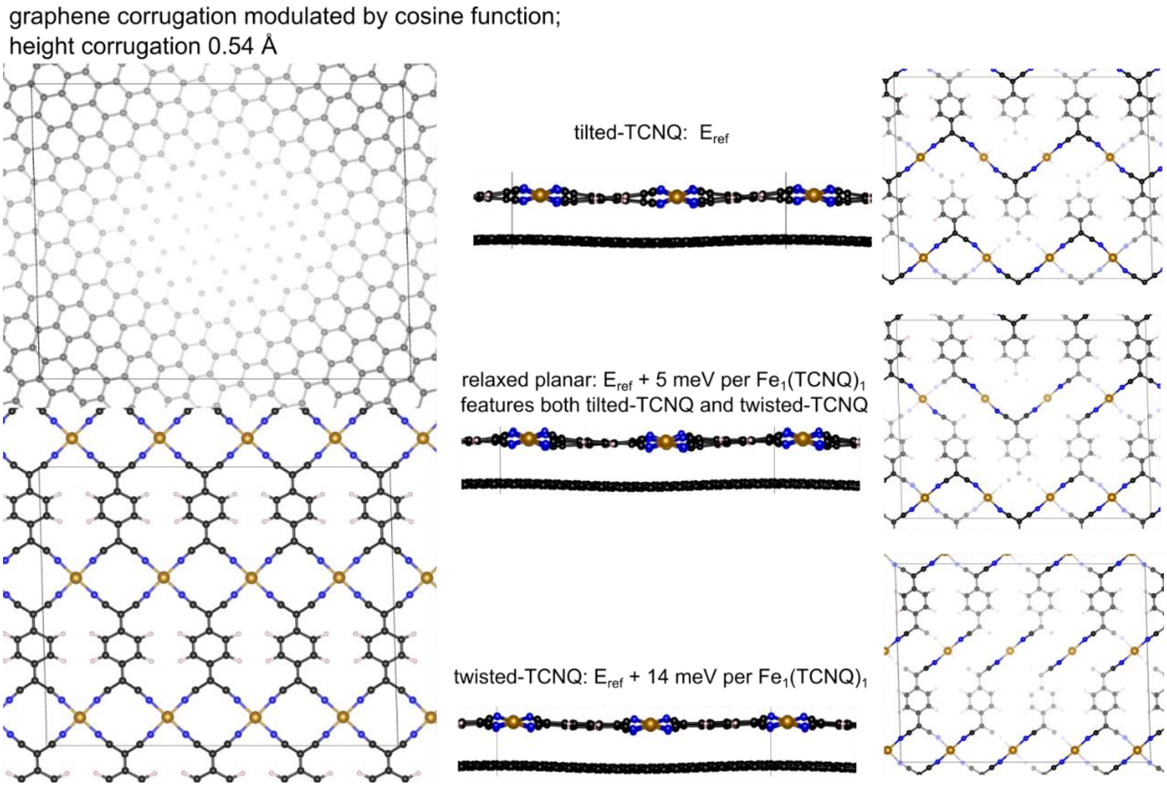

Figure S7: The computational unit cell of corrugated graphene sheet designed by modulating the carbon atom height by a cosine function. The Fe-TCNQ models relaxed atop this corrugated graphene sheet show the same stability trends as on planar graphene sheet.

5. Additional experimental data on Fe-TCNQ/Au

Figures S8 and S9 show LEEM analysis of Fe-TCNQ/Au(111). Bright field and dark field images in Figure S8 show the spatial distribution of the individual orientations at a micrometer scale. Similarly to the previously described Fe-TCNQ/graphene case,[1] the substrate step edges play an important role in the orientation preference of neighboring Fe-TCNQ islands. The biggest patches are aligned with the longer Fe-TCNQ axis parallel to the step edges. This is clearly observed in panel b, where the patches are up to several μm wide. In the other orientations, the patches dimensions are about 50-100 nm.

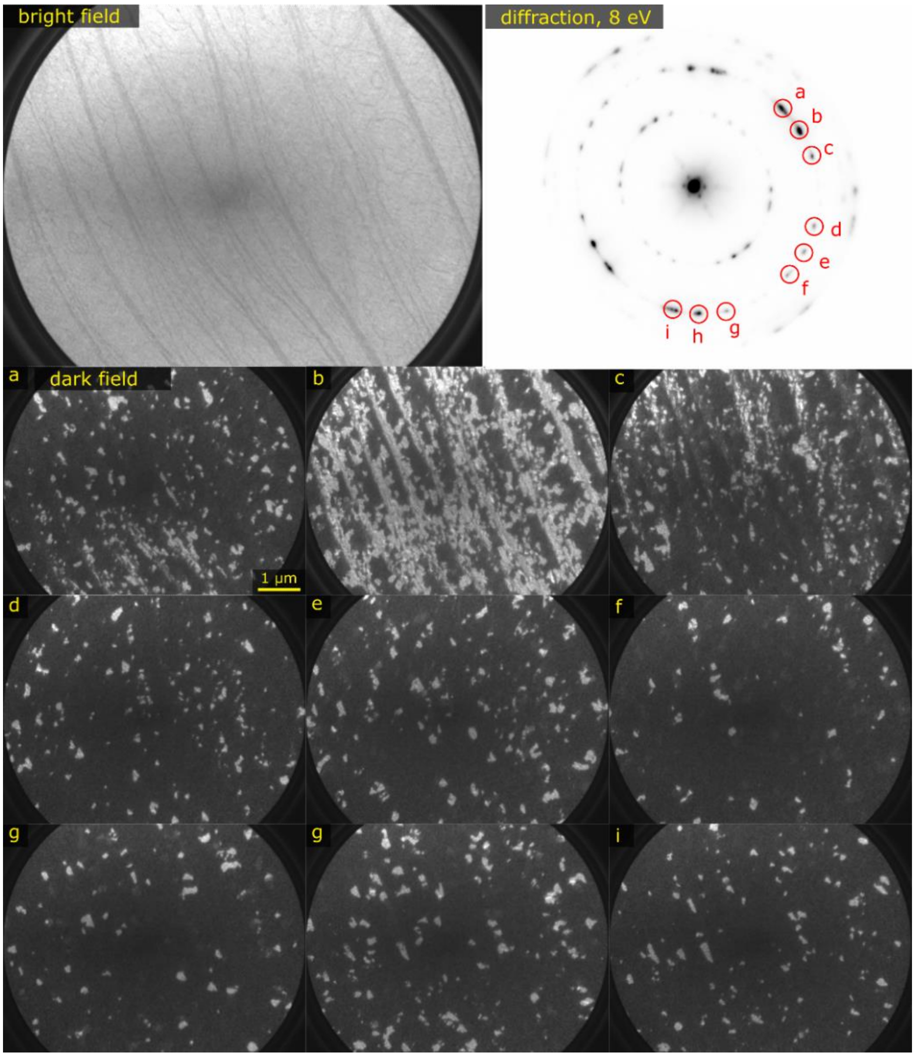

Figure S8: Bright field and dark field LEEM images of the Au-supported Fe-TCNQ network. Dark field panels (a-i) are projected from the diffraction spots labelled (a-i) in the diffraction pattern shown in top-right. Patches with dimensions in the 50-100 nm range are present in all the orientations, but by far the largest are observed in the orientation (b), where the dimension are in the µm range. This is most likely due to the fact that this Fe-TCNQ orientation aligns well with the majority direction of the Au(111) step edges, as seen in the bright field image. Previously, we have observed the same effect also on graphene-supported Fe-TCNQ. [1]

Figure S9 shows the comparison of experimental and simulated LEED patterns of Fe-TCNQ/Au(111). In the simulated pattern, the red spots correspond to an Fe-TCNQ orientation with the longer axis aligned with one of the Au(111) unit vectors. The yellow spots then correspond to orientations rotated by 13° from the Au(111) unit vectors. In panel B, the pattern is simulated with the Fe-TCNQ dimensions of (7.3 x 11.7) Å, which previously provided a reasonable agreement for Fe-TCNQ/graphene (although with a significant uncertainty due to the calibration only to the Gr/Ir moiré, and not the bulk substrate spots, Fig. S4C). This pattern reproduces the relative positions of the Fe-TCNQ well, but does not reproduce the position of the Fe-TCNQ pattern with respect to the Au substrate (disagreement highlighted by the green ovals in panel B). The relative positions of the Fe-TCNQ with respect to Au(111) are then much better reproduced by a smaller Fe-TCNQ unit cell of (6.6 x 10.8) Å. This may suggest that the Fe-TCNQ unit cell is compressed on Au(111), but one must take into account the significant uncertainty of the reference measurements on graphene. In any case, some compressive stress acting on the Fe-TCNQ is consistent with the fact that the Au(111) is clearly subjected to tensile stress under Fe-TCNQ, as evidenced by the STM images shown in the main text and in Figure S10 below.

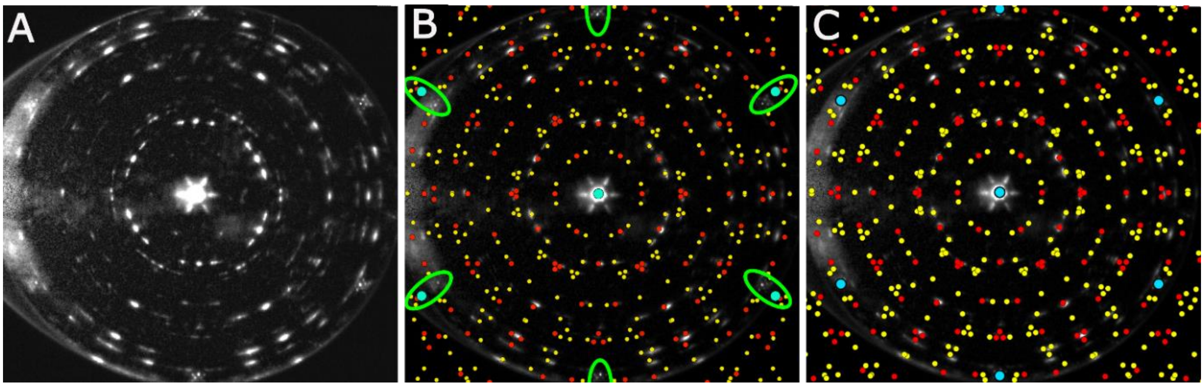

Figure S9: (A) Experimental LEED pattern acquired on Au(111)-supported Fe-TCNQ. B) The experimental LEED pattern overlaid with a pattern simulated for Fe-TCNQ dimensions of (7.3 x 11.7) Å, which previously showed reasonable agreement for graphene-supported Fe-TCNQ. This does not reproduce the relative position of the Fe-TCNQ spots and Au(111) spots, as highlighted by the green ovals. C) Experimental LEED pattern overlaid with a pattern simulated for Fe-TCNQ dimensions of (6.6 x 10.8) Å.

Figure S10 shows STM images of partial coverage of Fe-TCNQ on Au(111). The STM images like the one in panel A could be calibrated by the periodicity of the herringbone reconstruction on pristine Au(111) (63 Å). The comparison to the extended herringbone periodicity under Fe-TCNQ islands clearly indicates that the Fe-TCNQ induces tensile stress on the Au substrate, and the nearest neighbor distance can get extended by several pm. Panel B shows a detail of an alternative Fe-TCNQ phase which is common on Au(111), but was never observed on graphene. As the Fe-TCNQ synthesis protocol involves excessive dose of TCNQ, our tentative assumption is that this alternative phase might have different $Fe_x(TCNQ)_y$ stoichiometry, where the TCNQ ends not coordinated to the Fe adatom might bend towards the Au substrate, similarly to what was observed with F_4TCNQ/Au(111).[4] Nevertheless, further studies would be necessary to fully elucidate this structure and its properties.

Figure S10C shows a boundary between two well-ordered islands of Fe-TCNQ/Au(111). The individual protrusions between the well-ordered areas are most likely TCNQ molecules. It is reasonable to assume that these molecules are bound to metal atoms, because the synthesis protocol involves post-annealing to 340 °C, while TCNQ on pristine Au(111) desorbs already at 140 °C. One plausible explanation is that these excess disordered TCNQ molecules might be bound to native Au adatoms, which were pushed from under the Fe-TCNQ as the Au(111) got extended. Further study would be necessary to confirm this hypothesis. In any case, this is another one of Fe-TCNQ properties which are clearly different between Au(111)-supported and graphene-supported systems.

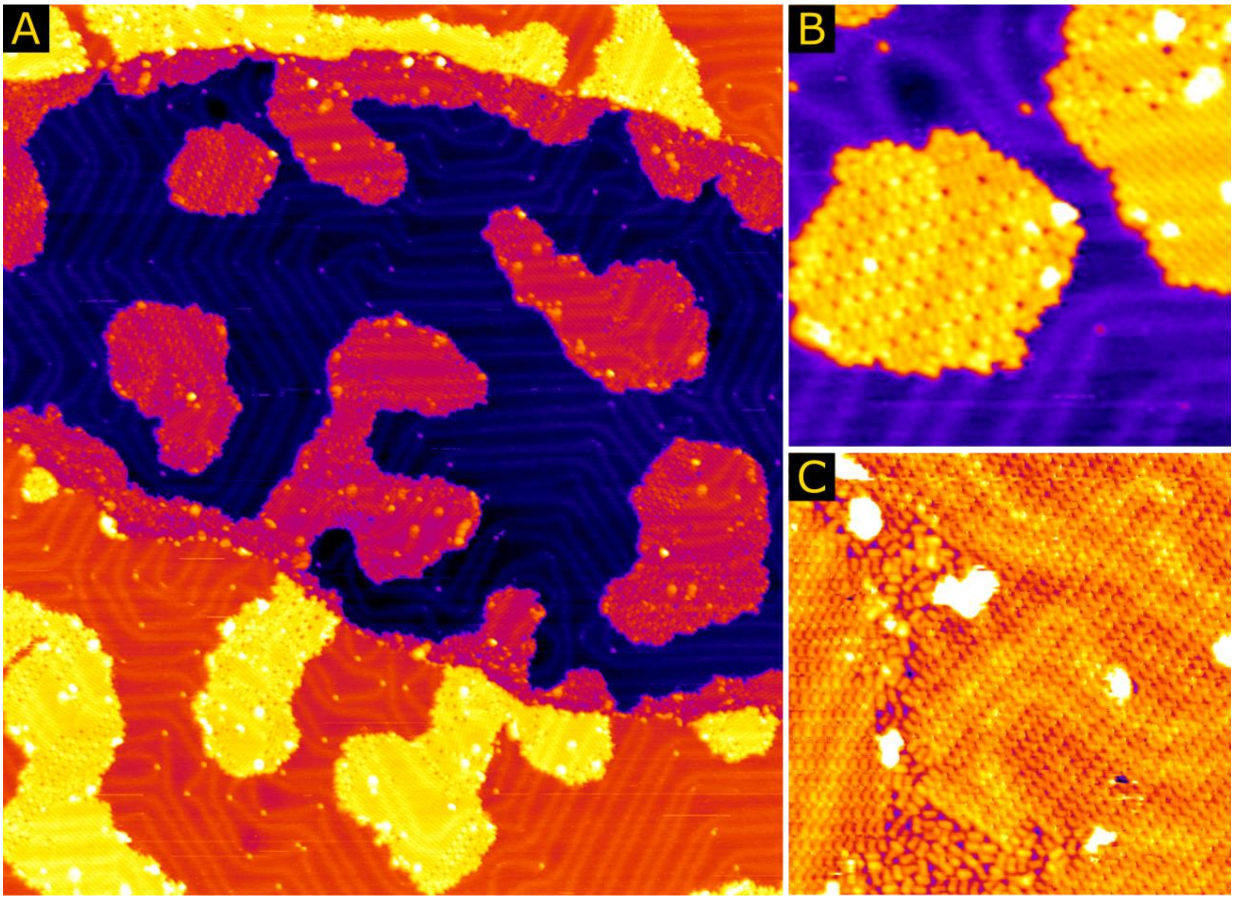

Figure S10: STM images of Fe-TCNQ/Au(111). A) STM of partial coverage of Fe-TCNQ on Au(111) allows to calibrate the image to the spacing of the herringbone reconstruction on the pristine Au(111). B) An alternative Fe-TCNQ phase which is commonly observed on Au(111), but not on graphene. C) The areas between well-ordered Fe-TCNQ patches on Au(111) are packed with additional species, likely TCNQ molecules possibly coordinated to Au adatoms.

6. Considered supercell for Fe-TCNQ/Au(111), comparison to experimental data

Figure S11 shows the supercell used for Au-supported models. The supercell is composed of four Au(111) layers thick substrate with the matrix notation $\begin{pmatrix} 2 & -7 \\ 6 & 0 \end{pmatrix}$ and the FeTCNQ layer with the matrix notation $\begin{pmatrix} 2 & 1 \\ -2 & 1 \end{pmatrix}$. This gold-supported Fe-TCNQ model is slightly compressed with respect to the gas-phase z-frozen planar model, but slightly extended with respect to the tilted-TCNQ gas phase model. Specifically, the match of the substrate and the Fe-TCNQ molecular layer yields small tensile strain compared to the gas-phase tilted-TCNQ model, by 1.6% in *a* direction, 4.4% *b* direction, and 2.6% angular strain. With respect to the z-frozen planar model in gas phase the strain is compressive, by -2.3% in *a* direction, -2.3% *b* direction, and 2.6% angular. The dimensions of all the considered models are listed in Table ST1 below. The angular alignment of the Fe-TCNQ with respect to the Au substrate in the supercell differs by $\approx 6°$ with respect to the most pronounced orientation identified in LEED (Fig. S9).

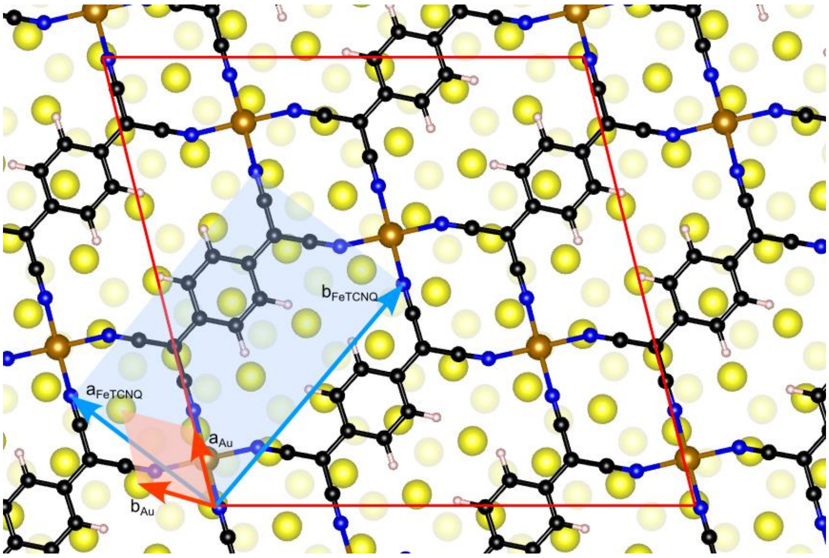

Figure S11: Computational unit cell used for Au(111)-supported Fe-TCNQ models.

7. STM simulations of different Fe-TCNQ/Au models

Figure S12 shows an experimental STM image acquired at sample bias $-1.3\ \mathrm{V}$ compared to computational STM simulations based on the considered Fe-TCNQ/Au models (bias $-1.4\ \mathrm{V}$). In the experimental image, the Fe-sites show no sign of elongation characteristic of twisted-TCNQ or tilted-TCNQ models (due to the different heights of the four cyano groups surrounding the Fe atom, see Figures 1,2 in the main text). Additionally, in the experimental STM images of Fe-TCNQ/Au, the apparent height of the cyano groups is several pm lower than that of the neighboring methyl groups; this suggests lower position of the Fe atom. These features are well reproduced by the STM simulation based on the planarized model. In contrast, the STM simulations based on the twisted-TCNQ and tilted-TCNQ show poor agreement with experiment.

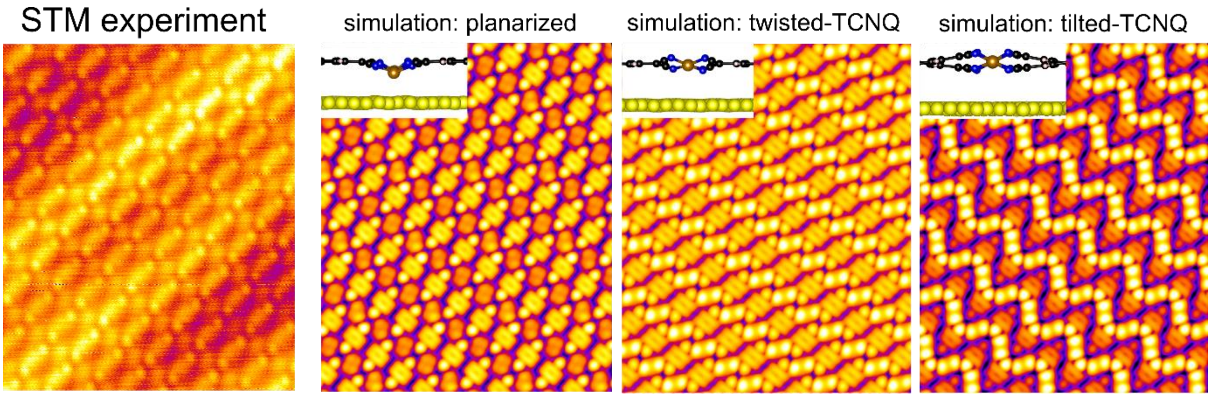

Figure S12: Experimental STM image of FeTCNQ/Au(111) compared to STM simulations based on planarized, twisted-TCNQ and tilted-TCNQ models. The best agreement is provided by the planarized model.

8. Fe-TCNQ lattice parameters from experiment and theory

Table ST1: Fe-TCNQ lattice parameters from computational models and experimental measurements.

Fe-TCNQ parameters on different substrates, acquired by different methods	*a* (Å)	*b* (Å)	θ (°)	Fe-support distance (Å)	Fe-N bond lengths (Å)
free-standing, tilted TCNQ (DFT)	7.0	10.7	90.0	-	2.03–2.03
free-standing, twisted TCNQ (DFT)	6.9	11.3	90.1	-	2.04–2.04
free-standing, z-frozen planar (DFT)	7.3	11.4	90.0	-	2.06–2.06
Graphene/Ir(111) (STM, LEED)	7.3 ± 0.5	11.7 ± 0.6	90 ± 3	-	-
Graphene (DFT, tilted TCNQ)	7.1	11.3	88.4	3.7	2.04–2.06
Graphene (DFT, twisted TCNQ)	7.1	11.3	88.4	3.7	2.04–2.05
Graphene (DFT, z-frozen planar)	7.1	11.3	88.4	3.4	2.02–2.04
Au(111) (STM)	7.0 ± 0.4	10.9 ± 0.4	90 ± 10	-	-
Au(111) (LEED)	6.6 ± 0.3	10.8 ± 0.4	90 ± 2	-	-
Au(111) (DFT, planarized with Fe closer to Au)	7.1	11.2	87.6	2.5	2.08–2.11
Au(111) (DFT, tilted-TCNQ)	7.1	11.2	87.6	3.7	2.03–2.06
Au(111) (DFT, twisted-TCNQ)	7.1	11.2	87.6	3.4	2.02–2.07

9. Stability of Fe-TCNQ in the gas phase

Table ST2: Computed stability of the different Fe-TCNQ models in the gas phase

Calculated stability of the Fe-TCNQ layer in the gas phase	ΔE / $Fe_1(TCNQ)_1$ (eV)
Tilted-TCNQ	0 (reference)
Tilted-TCNQ (alternative, see section 13 in the SI)	+0.001
Twisted-TCNQ	+0.216
z-frozen planar	+0.314

10. Stability of Fe-TCNQ molecular layer on graphene and on gold

We calculated stability of Fe-TCNQ on gold and on graphene following the equation

$$\Delta E = E_{\text{layer+substrate}} - (E_{\text{layer}} + E_{\text{substrate}}),$$

where the first term represents the total energy of the interacting system and the latter terms stand for the total energies of the separated counterparts. E_{layer} is the energy of the most-stable tetrahedral gas phase configuration calculated at the lattice constants that correspond to the substrate supercell.

Table ST3: Stability of the Fe-TCNQ models on graphene and on gold

Calculated stability of the Fe-TCNQ layer on different substrates	ΔE / $Fe_1(TCNQ)_1$ (eV)
Au(111) (planarized with Fe closer to Au)	-1.276
Au(111) (twisted-TCNQ)	-1.278
Au(111) (tilted-TCNQ)	-1.305
Graphene (z-frozen planar)	-1.010
Graphene (planarized with Fe closer to graphene)	unstable
Graphene (twisted-TCNQ)	-1.121
Graphene (tilted-TCNQ)	-1.142

11. The energy penalty associated with designing commensurate structure models

Our DFT methodology uses models with periodic boundary conditions, which require models with commensurate MOF/support structures. This inevitably leads to some level of strain. Here, we quantify how this strain affects the stability of the individual models. We have calculated the energy of the Fe-TCNQ models in the gas phase under the same lateral strain as in the supported models. The numbers in table below were calculated for each model following the equation

$$\Delta E = E_{\text{gas\_phase\_strained}} - E_{\text{gas\_phase\_unstrained}},$$

and thus represent the energy penalty associated with the supercell-induced lateral strain.

Table ST4: The energy penalty associated with designing commensurate structure models.

Energy penalty caused by fixing the lateral unit cell dimensions to those of Gr- and Au-supported superstructures	Unstrained (reference) ΔE / $Fe_1(TCNQ)_1$ (eV)	Gr-supercell dimensions ΔE / $Fe_1(TCNQ)_1$ (eV)	Au-supercell dimensions ΔE / $Fe_1(TCNQ)_1$ (eV)
z-frozen planar	0.000	0.093	0.142
Tilted-TCNQ	0.000	0.111	0.088
Twisted-TCNQ	0.000	0.022	0.028

On graphene supercell, the structure most disfavored by the lateral strain is the tilted-TCNQ structure. In other words, our DFT methodology underestimates the stability of tilted-TCNQ on graphene. This further supports our conclusions about the physical structure of Fe-TCNQ on graphene as given in the main text.

In our Fe-TCNQ/Au(111) supercell, the structure most disfavored by the lateral strain is the z-frozen planar (which is unstable anyway) followed by the tilted-TCNQ structure. Obviously, our method of energy comparison cannot be applied to the planarized structure with Fe drawn towards Au, which is only stabilized by the vdW interaction between Fe and Au, and thus is unstable in the gas phase. Nevertheless, this structure is fully consistent with our experimental data as well as with the previous studies of similar TCNQ-based structures, where the metal atoms are attracted to the gold supports.

12. The effect of lateral strain on the electronic structure of planarized FeTCNQ/Au

The evidence that the electronic structure of the tilted-TCNQ and twisted-TCNQ models on graphene and on Au(111) is not affected by the small lateral strain associated with the commensurate unit cell design is provided in Figure 4B,C,D,E in the main text, where very similar features are observed in tilted- and twisted-TCNQ models in the gas phase, on graphene and on Au(111).

For the case of planarized Fe-TCNQ structure on Au(111), we have tested the effect of lateral strain on the electronic structure by designing Fe-TCNQ/Au unit cells in which the Fe-TCNQ is slightly compressed or extended. The results are summarized in Figure S13. In a unit cell compressed by 1.3 % in *a* direction and 0.9 % in *b* direction, the features in the electronic structure are very similar as in the original model (Figure S13B). The same is observed for most Fe atoms in a structure extended by 2.5 % in *a* and 3.0 % in *b* (Figure S13C). Only 1 out of 4 Fe atoms in this extended structure was found to have fully occupied d_{z^2} orbital (not shown). We note that such extended structure is definitely not present in experiment: First, it is extended even more than a gas-phase planar structure, and second, the experimental data clearly indicate compression of Fe-TCNQ.

Based on this dataset we conclude that our conclusions about the electronic structure of various Fe-TCNQ models are not strongly affected by the lateral strain induced by the design of commensurate DFT models.

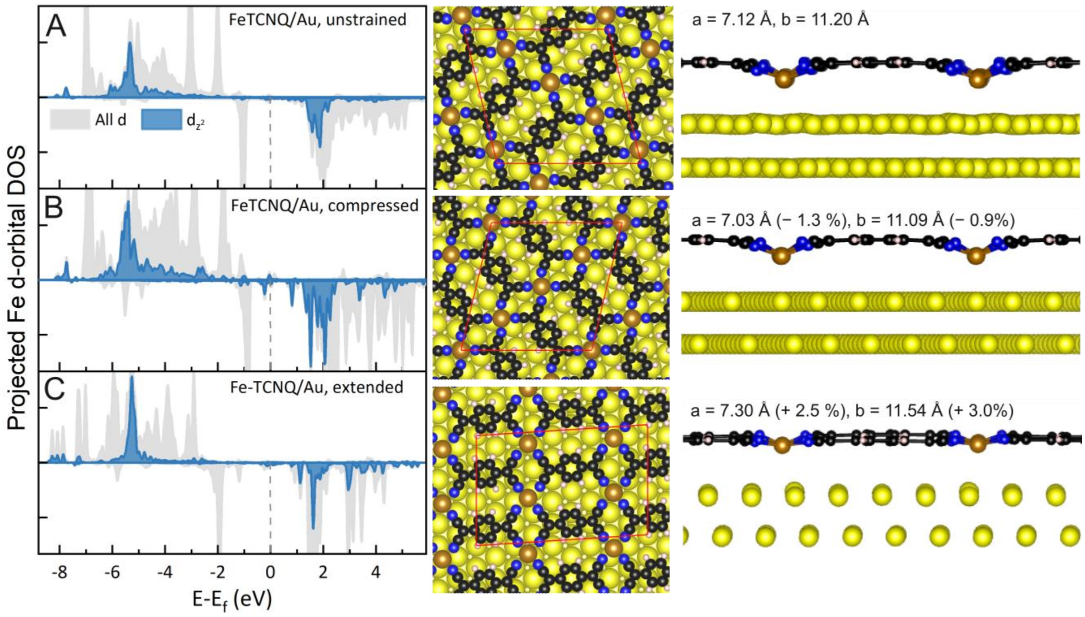

Figure S13: Density of states of the Fe d-orbitals in differently strained planarized Fe-TCNQ structures. A) the original model described in the main text. B) An alternative, compressed model. The electronic structure of the Fe d-orbitals is very similar to the original model. C) An alternative, extended model. In this model, 3 out of 4 Fe atoms have electronic structure very similar to the original model, only 1 out of 4 atoms has a fully occupied d_{z^2} orbital (not shown). However, such an extended structure is definitely not present in experiment.

13. Comparison of the two models for gas-phase tilted-TCNQ structure

In the gas-phase DFT model, we identify two models of tilted-FeTCNQ which are isoenergetic (within units of meV), but slightly differ in the structure and d-electron configuration (Figure S14). Specifically, the structure shown in panels A,C has slightly lower height corrugation of the N atoms. The electronic structure of the Fe atoms within this structure shows slightly less pronounced unoccupied d_{z^2} state at 1.3 eV (panel E). Despite the small differences between those two models, both follow the trends described in the main text.

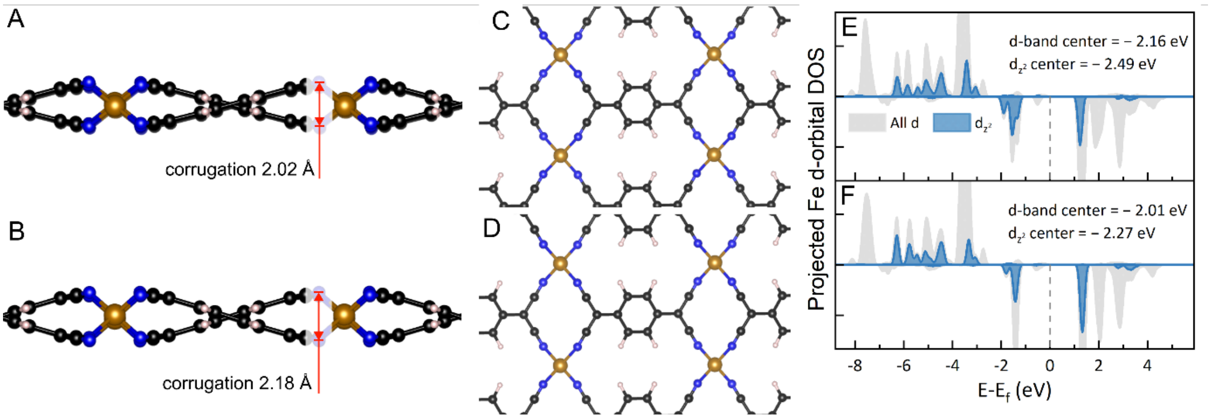

Figure S14: Comparison of two isoenergetic gas-phase tilted-FeTCNQ models. Panels (A,B) show the side view of the two structures, (C,D) top views and (E,F) the DOS plots of the Fe d-orbitals.

14. Spin-polarization of all the considered models

All the tested models are qualitatively similar in Fe charge state and magnetic moment, consistent with a Fe(II) d^6 cation in a high-spin S=2 quintet state. Low-spin and intermediate-spin models were tested and some converged to local energy minima. However, they were always energetically worse than models with S=2. Table ST5 contains explicit information of the spin-polarization of in the occupied d-orbitals (density of states of majority spin and minority spin states integrated up to Fermi level).

Table ST5: Spin-polarization of different Fe-TCNQ models.

Fe-TCNQ model	Majority spin (Fe d electrons)	Minority spin (Fe d electrons)	Total occupied (Fe d electrons)
free-standing, z-frozen planar (DFT)	4.6	1.0	5.6
free-standing, twisted TCNQ (DFT)	4.6	1.1	5.7
free-standing, tilted TCNQ (DFT)	4.6	1.1	5.7
Graphene/Ir(111), (DFT, tilted TCNQ)	4.4	1.0	5.4
Au(111) (DFT, tilted-TCNQ)	4.4	1.0	5.4
Au(111) (DFT, planarized with Fe closer to Au)	4.6	1.0	5.6

15. Comparison of the individual models to the UPS spectra

Figure S15 shows the experimental UPS difference spectra compared to the computed density of states of the individual Fe-TCNQ models on graphene and on gold supports (the plotted DOS is projected on all the orbitals within the Fe-TCNQ. The states corresponding to the support are not plotted). The total density of states differences between the individual models are quite subtle, but the best agreement is provided by the tilted-TCNQ model on graphene, and the planarized-TCNQ model on Au(111).

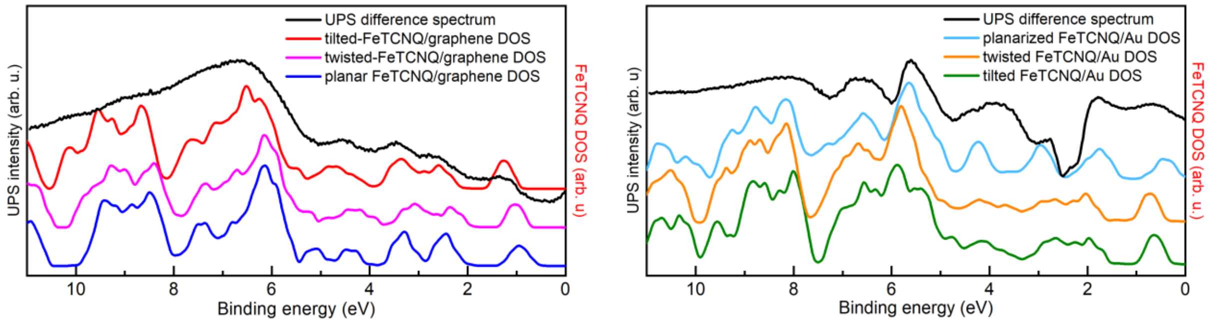

Figure S15: Experimental UPS difference spectra of Fe-TCNQ/graphene (left) and Fe-TCNQ/Au (right) compared to the DOS computations of the individual Fe-TCNQ models (planar, twisted, tilted-TCNQ).

16. Polar angle dependence of the UPS difference spectra

Figure S16 shows the experimental UPS difference spectra averaged over different ranges of polar angles. In the graphene-supported case, the same features are observed, but with varying intensities depending on the polar angle summation. In the Au-supported case the analysis is complicated by the dominant Au 5d bands. Thus, the difference spectrum shows strong dependence on the polar angle summation.

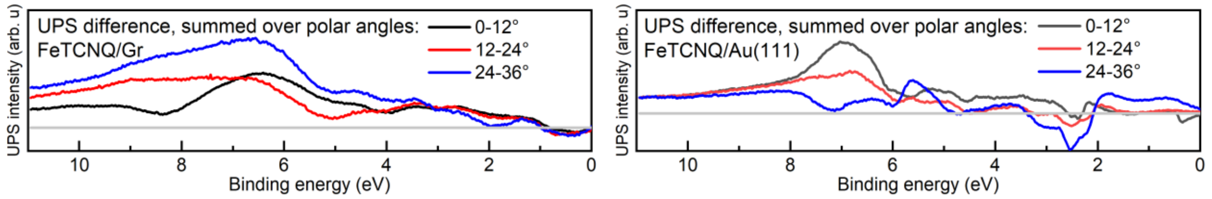

Figure S16: Experimental UPS difference spectra summed over different ranges of polar angles.

17. Additional STM images of Fe-TCNQ/Au before and after TCNQ adsorption

Figure S17 shows STM images of the Fe-TCNQ/Au sample before and after deposition of additional TCNQ. The freshly prepared sample (images on the left side of Figure S17) features many areas of disordered Fe-TCNQ near domain boundaries of well-ordered Fe-TCNQ. After TCNQ deposition and heating to 65 °C, it seems that most of the additional TCNQ is stabilized at the areas of the disordered Fe-TCNQ; not directly above well-ordered Fe-TCNQ.

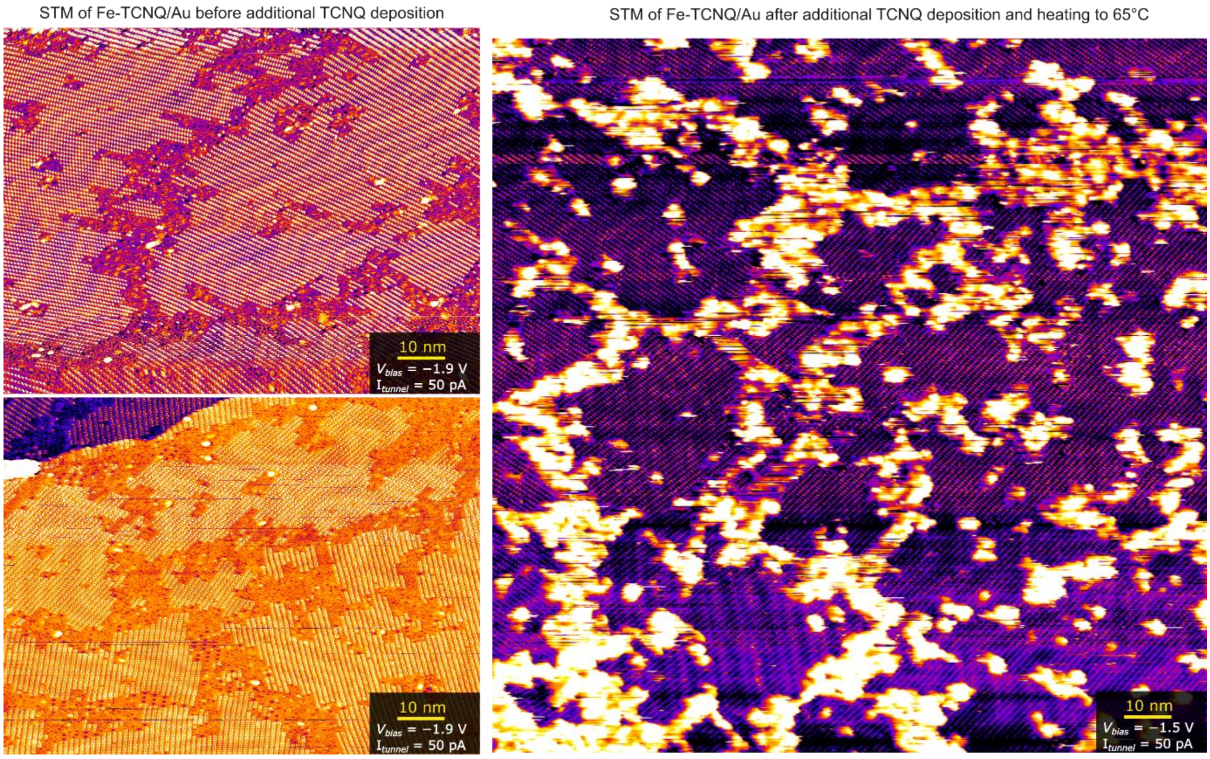

Figure S17: STM images of Fe-TCNQ as prepared (left) and after additional TCNQ deposition and heating to 65 °C. The comparison reveals that most of the TCNQ adsorbed at 65 °C most likely resides on the areas of disordered Fe-TCNQ (darker in the images of freshly prepared sample).

18. DFT models of monolayer TCNQ atop Fe-TCNQ/Au

Figure S18 shows different DFT models considered for the monolayer coverage of TCNQ atop Fe-TCNQ/Au(111). These models differ in the lateral positioning of the monolayer and the support (A vs. B) and in the intermolecular spacing in the top layer (A,B vs. C). In models A,B, the intermolecular spacing of the top layer is based on that of the underlying Fe-TCNQ layer, in model C it is based on simulations of a free-standing TCNQ monolayer. Model B shows that a chemical bond between the layers can be formed when a CN group resides directly above the Fe atom. Such occurrences are however scarce (in this model, only 1 out of 16 -CN groups is chemically interacting). Importantly, all the considered models feature very similar levels of vertical charge transfer, leading to very similar computational work function changes.

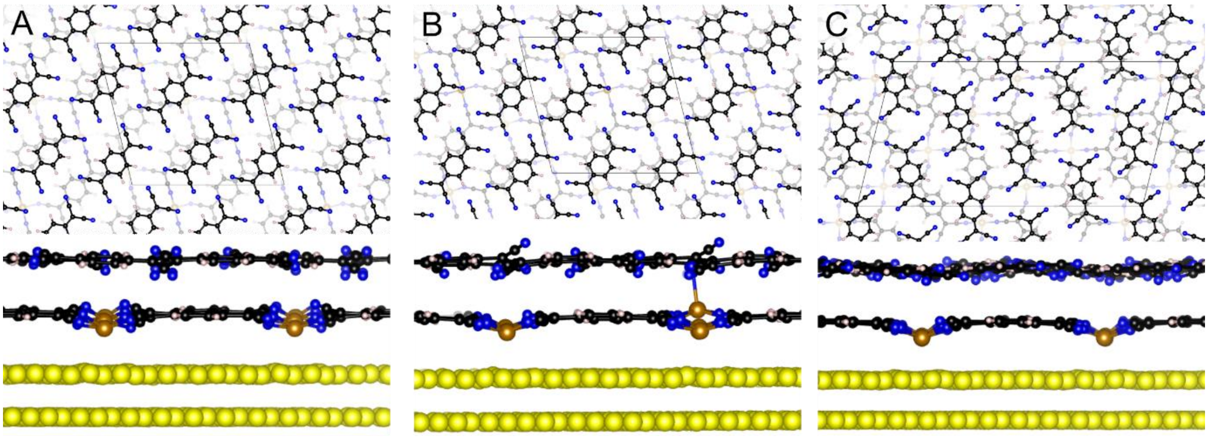

Figure S18: Different DFT models considered for the monolayer coverage of TCNQ atop Fe-TCNQ/Au(111). The work function differences induced by the presence of top-layer TCNQ are: (A): ΔW = 0.22 eV, (B) ΔW = 0.31 eV, (C) ΔW = 0.32 eV.

19. Description of Supplemental movie 1

Supplemental movie 1 shows LEED patterns of Fe-TCNQ/Au(111) at electron energies between 5 eV and 40 eV. The energy step between frames is 0.5 eV.

References

(1) Jakub, Z.; Kurowská, A.; Herich, O.; Černá, L.; Kormoš, L.; Shahsavar, A.; Procházka, P.; Čechal, J. Remarkably Stable Metal–Organic Frameworks on an Inert Substrate: M-TCNQ on Graphene (M = Ni, Fe, Mn). *Nanoscale* **2022**, *14* (26), 9507–9515.

(2) Altenburg, S. J.; Berndt, R. Local Work Function and STM Tip-Induced Distortion of Graphene on Ir(111). *New. J. Phys.* **2014**, *16* (5), 53036.

(3) Hämäläinen, S. K.; Boneschanscher, M. P.; Jacobse, P. H.; Swart, I.; Pussi, K.; Moritz, W.; Lahtinen, J.; Liljeroth, P.; Sainio, J. Structure and Local Variations of the Graphene Moiré on Ir(111). *Phys. Rev. B* **2013**, *88* (20), 201406.

(4) Mousley, P. J.; Rochford, L. A.; Ryan, P. T. P. P.; Blowey, P.; Lawrence, J.; Duncan, D. A.; Hussain, H.; Sohail, B.; Lee, T.-L. L.; Bell, G. R.; Costantini, G.; Maurer, R. J.; Nicklin, C.; Woodruff, D. P. Direct Experimental Evidence for Substrate Adatom Incorporation into a Molecular Overlayer. *J. Phys. Chem. C* **2022**, *126* (16), 7346–7355.
